# An Experimental Benchmark for the Barrier Height of an On-Surface Reaction: Hydrogen Oxidation on Pt(332)

**DOI:** 10.1021/jacs.5c12862

**Published:** 2025-10-03

**Authors:** Florian Nitz, Stefan Hörandl, Michael Schwarzer, Theofanis Kitsopoulos, Daniel J. Auerbach, Alec M. Wodtke

**Affiliations:** † Institute for Physical Chemistry, Georg-August University of Goettingen, Tammannstraße 6, 37077 Goettingen, Germany; ‡ Department of Dynamics at Surfaces, Max Planck Institute for Multidisciplinary Sciences, Am Fassberg 11, 37077 Goettingen, Germany; § School of Mathematics and Natural Sciences, University of Southern Mississippi, Hattiesburg, Mississippi 39406, United States; ∥ International Center for Advanced Studies of Energy Conversion, Georg-August University of Goettingen, Tammannstraße 6, 37077 Goettingen, Germany

## Abstract

Developing predictive theories for the rates of reactions between surface-bound molecules is a central challenge to understanding many important phenomena including: heterogeneous catalysis, electrocatalysis, nanofabrication, and corrosion. To meet this challenge, chemically accurate benchmarks to test theoretically derived reaction rates and barrier heights are essential, but few exist. Here, we determine from experiment an accurate zero-point-energy corrected barrier height, 0.76 ± 0.03, for the reaction O* + H* → OH* occurring at atomic Pt B-type step sites, the rate limiting step of hydrogen oxidation on Pt. This experimental benchmark agrees with density functional theory (DFT) predictions made at the level of the generalized gradient approximation (GGA) for five different functionals, exhibiting a mean absolute error (MAE) of 25 meV. This is far better agreement than commonly expected for this level of theory. We speculate that this level of agreement may be a common feature for reactions that involve only species adsorbed on surfaces.

## Graphical abstract

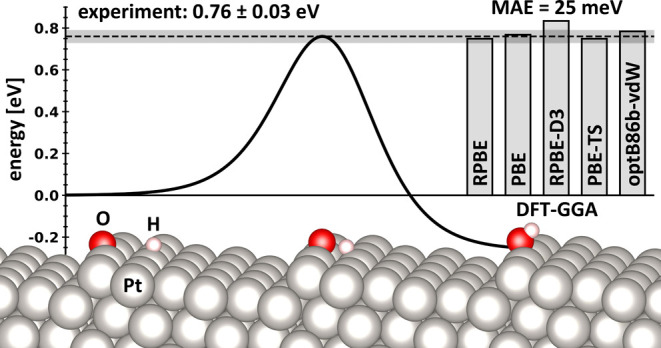

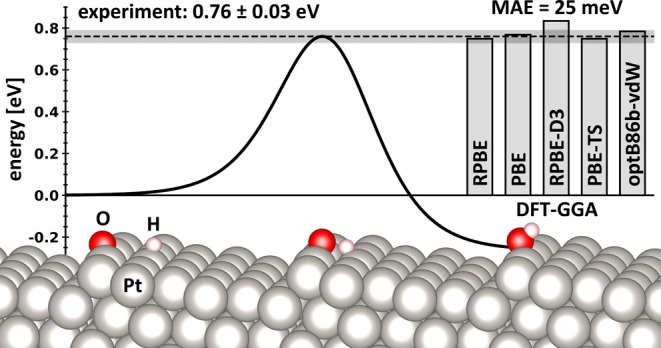

## 1 Introduction

In heterogeneous catalysis, stable molecules first bind to a catalytic surface and become chemically activated. These adsorbates subsequently react with one another in a complex network of on-surface thermal reactions leading to formation and desorption of products. On-surface reactions often determine the turnover frequency and the product selectivity of a catalyst;^1–3^ but sadly, elementary on-surface reactions are extraordinarily challenging to observe directly. Consequently, a predictive first-principles theory of their rates would be invaluable in catalyst design and optimization, and would also advance studies in electrocatalysis, corrosion, and nanofabrication.

On-surface reaction rates can be predicted using transition state theory (TST), an approach which requires efficient and accurate ways to compute the energies and entropies of adsorbates residing at specific stationary points of the underlying potential energy surface (PES), those reflecting the reactant’s initial state and the reaction’s transition state. Density functional theory (DFT) at the level of the generalized gradient approximation (GGA)^4–6^ has become the most widely used approach to compute these quantities;^2,7–11^ but up to now, the lack of sufficiently accurate benchmarks for on-surface reactions has made it impossible to test the accuracy of this approach. On the other hand, benchmarking studies involving the adsorption and desorption of small molecules at transition metal surfaces, which we henceforth refer to as gas-surface reactions, are plentiful albeit comparisons are less than encouraging. For example, benchmarks exist for the barrier heights of dissociative adsorption of H_2_ on Cu(111),^12,13^ O_2_ on Al(111)^14,15^ and CH_4_ on Pt(111).^13,16^ In addition, accurate adsorption energies are available from single crystal adsorption calorimetry (SCAC).^17^ When DFT-GGA predictions are compared to these established benchmarks, mean absolute errors of 0.4 eV are found^18–20^ with the choice of the exchange-correlation functional altering the predicted energies by up to 0.8 eV.^20^ These errors in the barrier height lead to very large errors in the rate, due to the exponential scaling of reaction rate with barrier height. These considerations lead one to doubt the reliability of theoretically predicted rates of elementary surface reactions involved in heterogeneous catalysis.

One might be so bold, however, as to hypothesize that the errors in computed barrier heights for on-surface reactions may be smaller than those found for gas-surface reactions. GGA functionals that accurately describe both gas-phase and solid-state systems remain to be realized.^21–25^ It is therefore not surprising that DFT-GGA derived barrier heights of gas-surface reactions are strongly functional dependent. In contrast, barrier height predictions for on-surface reactions are a problem purely of the solid state and one might, therefore, suppose that GGA functionals perform better for these purely on-surface reactions than they do for gas-surface reactions. Unfortunately, testing this hypothesis requires accurate benchmarks for barrier heights of on-surface reactions, of which there are none of sufficient accuracy.

In this work we provide a new experimental benchmark for the barrier of an on-surface reaction that to our knowledge is the first to provide chemical accuracy (1 kcal/mol or 43 meV). The benchmark is based on thermal rate constants measured with velocity-resolved kinetics (VRK).^26^ Recent improvements of VRK using a high-repetition rate (HRR) laser,^27^ provide a much higher experimental duty cycle and allow kinetic data to be obtained over a wide temperature range and at much lower adsorbate coverages.^27^ This minimizes the effect of lateral interactions between adsorbates and enables more rigorous comparisons to ab initio kinetics based on DFT-GGA input. Specifically, we have applied HRR-VRK to measure the thermal rates of hydrogen and deuterium oxidation on a stepped Pt(332) surface for low adsorbate coverages (<6 × 10^–3^ ML) from 473 to 673 K. We find that a microkinetic model based on DFT-GGA input to harmonic transition state theory (TST) accurately reproduces the observed kinetics, revealing that the rate-determining step (RDS) is the reaction O* + H*/D* → OH*/OD* occurring at B-type Pt step sites. This approach to ab initio kinetics reproduces the experimentally derived rate constants of the RDS within a factor of 2.5 and allows us to deduce an optimized value for the barrier height *E*_0_ of the reaction. We compare this experimentally derived *E*_0_ to barrier heights obtained from five GGA functionals and find a mean absolute error of 25 meV. These findings provide a chemically accurate benchmark for a barrier height of an on-surface reaction and suggest that DFT employed at the level of the GGA may perform markedly better for on-surface reactions than it does for gas-surface reactions.

## 2 Methods

### 2.1 Experimental

We use the HRR-VRK method to study transient kinetics of the hydrogen oxidation reaction on Pt(332). Details of the apparatus and the detection scheme have been described previously.^27^ In the following, we give a brief summary of the sample preparation and the procedures used in the current experiments.

All measurements are done using a round 1 cm diameter 2 mm thick Pt single crystal (MaTeck GmbH) with two facets—Pt(111) and Pt(332). The manufacturer specifies a cut angle accuracy of <0.1° for this crystal. In this study, we conduct experiments on the Pt(332) facet, which, based on the cut angle, consists of (16.7 ± 0.1)% B-type steps separated by (111) terraces. Prior to the experiment, the surface is cleaned by Ar^+^-ion sputtering (3 keV) for 20 min, followed by 20 min annealing in 10^–7^ mbar oxygen at 1100 K and then annealing in UHV at 1200 K for another 20 min. Surface cleanliness is verified with Auger electron spectroscopy. The sample temperature is controlled by radiant heating from a tungsten filament mounted behind the crystal and measured using a type-K thermocouple.

We precover the crystal with H or D by leaking molecular hydrogen or deuterium into the UHV chamber—the pressure is held constant during the experiment and monitored with a hot-cathode ionization gauge (Granville-Phillips 370 Stabil-Ion, MKS). Hydrogen and deuterium surface concentrations [H*] and [D*] are computed using a Langmuir isotherm and controlled at values from 0.1 × 10^12^ to 9 × 10^12^ atoms/cm^2^. Section S1 of the Supporting Information (SI) gives further details on the coverage determination and an empirical formula that accurately reproduces the Langmuir isotherm.

The hydrogen oxidation reaction is initiated by a supersonic molecular beam of pure O_2_ (50 μs FWHM pulse duration, 2 × 10^11^ molecules/pulse, 3 mm diameter at the Pt surface measured at 5% of the peak flux) incident at an angle of 30° with respect to the surface normal at repetition rates from 5 to 20 Hz. The absence of impurities in the molecular beam is verified using mass spectrometry.

Product and directly scattered molecules leave the surface and are ionized by nonresonant multiphoton ionization using a Yb-slab laser (Light Conversion, Carbide CB3–40W, 1030 nm, pulse duration 29 fs fwhm, 30 W, 100 kHz repetition rate), which is aligned parallel to the surface at a distance of 25 mm. Ionization occurs in a homogeneous electric field directed normal to the plane defined by the laser beam and the surface normal. Ions are accelerated along this direction retaining the other components of their velocities and are detected by a combined Chevron MCP/phosphor scintillator detector 46 cm from the ionization laser beam. Light pulses emitted by the scintillator pass through a window and are recorded outside the UHV chamber using an event camera based on CERN Timepix3 technology (Amsterdam Scientific Instruments TPX3CAM).

For each detected ion, the ion time-of flight (related to the mass-to-charge ratio), the time since the most recent molecular beam pulse (related to the reaction time) and the pixel positions (related to the in-plane velocity) are recorded. This allows profiles of product flux vs reaction time to be simultaneously recorded for all species emitted from the surface. We denote these profiles as “kinetic traces”. More details can be found elsewhere.^27^

### 2.2 Computational

The DFT calculations are performed using VASP 6.4.3.^28–30^ In all calculations, the plane wave cutoff is set to 500 eV, partial electronic occupancies are modeled using the Methfessel–Paxton^31^ (*N* = 2) scheme using a width of σ = 0.15 eV. The *k*-point grid of the Brillouin zone is sampled using a (3 × 3 × 1) mesh according to the scheme proposed by Monkhorst and Pack.^32^ To study the effect of the GGA exchange correlation functional, we perform calculations with the RPBE^33^ and PBE^5^ functionals, as well as their van der Waals corrected variants RPBE-D3 ^34^ and PBE-TS^35^ and one nonlocal van der Waals functional, optB86b-vdW.^36^

We use a slab model to represent the Pt(332) surface using a (4 × 1) unit cell with 24 surface atoms and 4 layers, the bottom layer is fixed to the bulk positions using optimized lattice constants (see SI Section S2). Positions of the other atoms are relaxed for all structures using the conjugate gradient algorithm until norms of their forces are smaller than 0.02 eV/Å. During this procedure, electronic energy optimization is stopped if the total free energy changes between two steps by less than 10^–5^ eV. A vacuum of 20 Å is introduced to the simulation cell to prevent interactions of the atoms with their periodic images perpendicular to the surface.

We use the climbing image nudged elastic band method^37^ to determine minimum energy paths between reactant and products. To calculate vibrational frequencies of adsorbates, we displace all adsorbate atoms by ±0.015 Å to construct and solve the Hessian matrix. During this procedure, the convergence criterion for the electronic loop is decreased to 10^–6^ eV.

### 2.3 Rate Constants from Transition State Theory

To compute thermal rate constants *k* for reactions between adsorbates A* and B*, we use transition state theory (TST), see eq 1. This requires knowing the zero-point energy (ZPE) corrected reaction barrier height *E*_0_ of the reaction and the unit cell area normalized partition functions of the transition state *Q* ^‡^ and the reactants *Q*_A*_, *Q*_B*_. These input parameters can be obtained from DFT calculations.

1 $$k=\frac{k_{B}T}{h}\frac{Q^{‡}}{Q_{A^{*}}Q_{B^{*}}}\exp\left(-\frac{E_{0}}{k_{B}T}\right)$$

The climbing image nudged elastic band method is used to determine classical barrier heights and we apply harmonic frequency calculations to obtain *E*_0_. If not stated otherwise, the partition functions of the adsorbates are based on a modified version of the harmonic oscillator partition function *Q*_HO_ given in eq 2,^38^ which accounts for multiple adsorption sites within the Pt(332) unit cell.

$$Q_{\mathrm{HO}}=\frac{1}{A_{\mathrm{cell}}}\overset{M}{\underset{j=1}{\sum }}\left[\exp\left(-\frac{E_{j}-E_{j=1}}{k_{B}T}\right)\overset{N_{j}}{\underset{i=1}{\prod }}{\left(1-\exp\left(-\frac{hv_{\mathrm{ij}}}{k_{B}T}\right)\right)}^{-1}\right]$$ 2

where *A*_cell_ is the area of the unit cell. We first compute *N*_*j*_ DFT frequencies *v*_*ij*_ of the adsorbates for all relevant adsorption sites, from which we obtain site-specific harmonic oscillator partition functions. *Q*_HO_ is computed from a temperature-dependent Boltzmann-weighted sum of these site-specific contributions over *M* adsorption sites. In this procedure, the Boltzmann weight is calculated based on the ZPE-corrected energy *E*_*j*_ – *E*_*j*=1_ of an adsorption site relative to the most stable adsorption site (*j* = 1), see also eq 2. We include all adsorption sites with *E*_*j*_ – *E*_*j*=1_ ≤ 0.5 eV to ensure sufficiently converged results.

Transition state partition functions *Q* ^‡^ are also calculated using eq 2, by using values of *v* representative of the real vibrational frequencies of the transition state. Usually, transition states for other adsorbate configurations lie significantly higher in energy, and in these cases the summation reduces to *M* = 1, corresponding to a single harmonic oscillator partition function.

Applying eq 2 to compute the partition function of the hydrogen atom *Q*_H*_ would neglect its wave nature, leading to significant errors.^39^ Instead, we use a previously introduced Quantum Potential Energy Sampling (QPES) method,^39^ that computes a fully quantum mechanical in-plane partition function of the hydrogen atom obtained by solving the Schrödinger equation in the in-plane potential energy surface. The result rigorously accounts for anharmonicity of the potential, delocalization and tunneling. The out-of-plane motion is treated separately using site-specific quantum harmonic oscillator partition functions. For more details of this procedure, the reader is referred to ref 39.

## 3 Results

We measured the time profile (kinetic trace) of the water formed in the hydrogen oxidation reaction initiated by a pulse of O_2_ incident on a Pt(332) surface that is precovered with H or D atoms. Figure 1(a) shows representative kinetic traces for D_2_O formation for 5 values of the D atom surface concentration, [D*], measured at a surface temperature of *T* = 573 K. The kinetic traces can be accurately fitted using a single exponential decay model (red line in Figure 1(a)) characterized by three parameters; an amplitude converting the simulated rate to the water signal seen in the experiment, a time shift (≤0.1 ms) to correct for the arrival time of the O_2_ beam and a first order decay rate constant *k*_eff_. These three parameters are fitted to each kinetic trace independently. The effective decay rate constant *k*_eff_ is directly proportional to [D*], as shown in the inset of panel (a). Panel (b) shows plots of *k*_eff_ vs [D*] for a range of surface temperatures between 473 and 673 K. An equivalent plot for the experiment with H_2_ gas inside the UHV chamber is shown in Section S3 of the SI.

**Figure 1:**
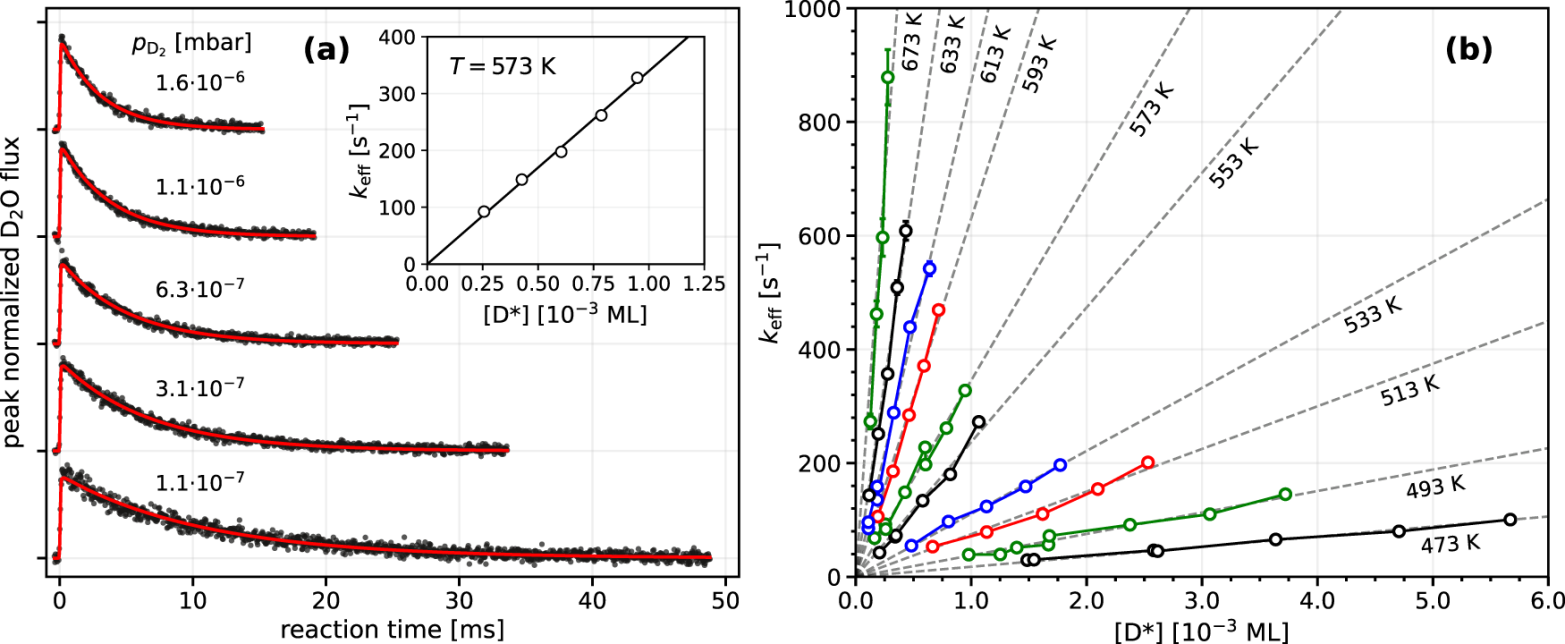
Kinetics of D_2_ oxidation on Pt(332). (a) The experimental D_2_O flux (black points) is fitted with a single exponential model (red line). Data is shown for five different D_2_ pressures inside the UHV chamber and for a surface temperature of 573 K. From each fit, we extract an effective decay rate constant *k*_eff_ which is proportional to the D atom surface coverage, [D*]. (b) The procedure is repeated for the indicated surface temperatures. Dashed lines are linear fits with zero intercepts, solid lines are only drawn to guide the eye. The error bars indicate 2σ-uncertainty intervals.

The exponential decay of the water formation rate indicates that the reaction is occurring under pseudo-first order reaction conditions, characterized by an approximately constant deuterium atom surface concentration that is maintained by the continuous and rapid adsorption and desorption of gas phase D_2_ present before and during the experiment. A simulation based on the calibrated flux and diameter of the O_2_ molecular beam and the measured reaction rates confirms this is the case.

The linear dependence of *k*_eff_ on [D*] allows us to conclude that D* is involved in the rate-determining step of the water formation reaction on Pt. To understand the significance of this result, it is useful to consider the elementary surface reactions that could lead to water formation. Previous work has suggested that hydrogen oxidation on Pt follows a Langmuir–Hinshelwood mechanism^40–44^ via the elementary reactions 3 to 5.

3 $$O^{*}+H^{*}\overset{k_{1}}{\to }{\mathrm{OH}}^{*}$$

4 $${\mathrm{OH}}^{*}+H^{*}\overset{k_{2}}{\to }H_{2}O^{*}$$

5 $$2{\mathrm{OH}}^{*}\overset{k_{3}}{\to }H_{2}O^{*}+O^{*}$$

We can immediately rule out the OH* disproportionation reaction 5 as rate limiting under the conditions of our experiment, as this reaction would result in kinetics that are independent of the hydrogen atom concentration. However, the experiment alone does not allow us to decide whether reaction 3 or 4 is rate limiting, so we turn to theory. Specifically, we develop a microkinetic model to simulate deuterium oxidation rates under the conditions of the VRK experiments based on TST rate constants derived from DFT calculations (both PBE and RPBE) for all three reactions occurring at atomic step sites.

The justification for assuming that reactions happen exclusively at step sites is twofold: First, our DFT calculations show that O* and OH* are stabilized at step sites compared to terrace sites by 0.28 and 0.54 eV, respectively. This is the case for both PBE- and RPBE-based calculations. Second, steps are more reactive toward OH* formation than (111) terraces. This enhanced reactivity can be qualitatively understood based on a Brønsted–Evans–Polanyi relationship;^45^ the increased reaction exoergicity of OH* formation at steps compared to terraces implies a lower OH* formation barrier for the reaction at steps. Details about barrier heights and partition function calculations are shown in the SI Section S4 for reaction 3, and in Section S5 for reactions 4 and 5. The construction and numerical solution of the microkinetic model is explained in SI Section S6.1.

Figure 2 shows representative comparisons of the predictions of this microkinetic model to experimentally observed D_2_O formation rates for three surface temperatures and several values of [D*]. The model’s predictions are in good agreement with experiment for both functionals; in fact, excellent agreement is obtained if the prefactor of *k*_1_ is slightly reduced while keeping the barrier height unchanged. This is shown by the red solid line, which is based on the PBE model with *k*_1_ × 0.54. A similar kinetic model assuming reactions occur at terrace sites—shown by the dashed lines—does not reproduce the experiment’s observations. Here we assume that the (111) terraces of Pt(332) can be described by a previously developed model for Pt(111).^42^

**Figure 2:**
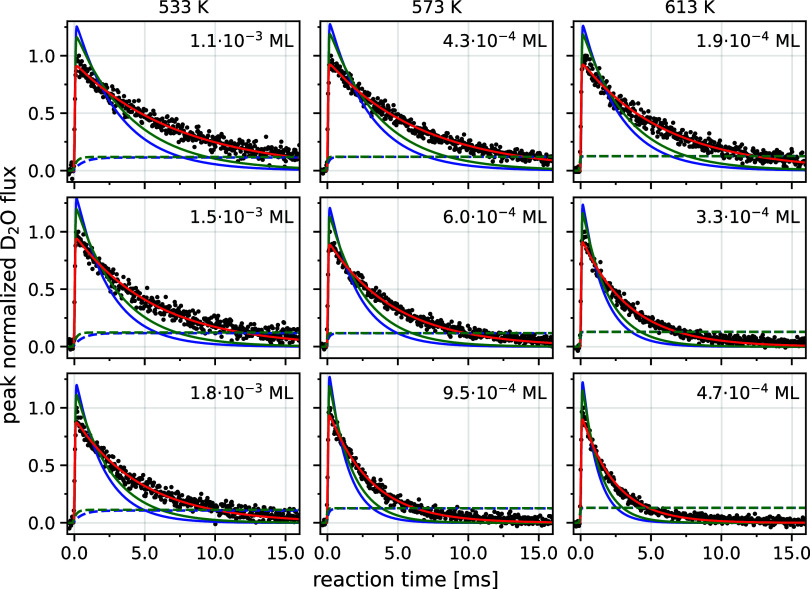
Simulated D_2_O formation rates (lines) in comparison to the experiment (points). The microkinetic model is shown with rate constants computed from RPBE (blue) and PBE (green). Solid lines are rate simulations at Pt(332) step sites, predictions for terraces are shown as dashed lines. The red solid line is based on the PBE model at step sites with an adjusted O* + D* → OD* prefactor (×0.54). Steady state deuterium coverages are given in each panel. Surface temperatures, given at the top of the columns, apply to all panels within a column. Data is peak normalized so that the amplitude of the model has been optimized for best agreement with the data.

Using the degree of rate control method to draw insights from this now experimentally validated rate model, we find that reaction 3 is the RDS and reactions 4 and 5 do not influence the observed kinetics. For details on this analysis see SI Section S6.2. While the model can be tuned to reproduce the experimental data with reaction 4 as the RDS, this requires decreasing the O* + H* reaction barrier by at least 0.25 eV and increasing the OH* disproportionation barrier by at least 0.80 eV—adjustments that exceed typical DFT errors. This strongly suggests that reaction 4 is not kinetically relevant under our conditions. Furthermore, since reaction 5 is significantly faster than the RDS, the water formation rate can be expressed simply using a steady state approximation of the OH* intermediate, see eqs 6 to 8.

6 $$\frac{d\left[{\mathrm{OH}}^{*}\right]}{dt}=k_{1}\left[O^{*}\right]\left[H^{*}\right]-2k_{3}{\left[{\mathrm{OH}}^{*}\right]}^{2}=0$$

7 $${\left[{\mathrm{OH}}^{*}\right]}^{2}=\frac{k_{1}}{2k_{3}}[O^{*}]\left[H^{*}\right]$$

$$\frac{d\left[H_{2}O^{*}\right]}{dt}=k_{3}{\left[{\mathrm{OH}}^{*}\right]}^{2}=\underset{k_{\mathrm{eff}}}{\underset{︸}{\frac{k_{1}}{2}\left[H^{*}\;\right]}}\left[O^{*}\right]$$ 8

In the VRK experiments described above, a constant hydrogen (or deuterium) atom surface concentration establishes pseudo-first order water formation rates. Under these conditions, the effective decay rate constant *k*_eff_ relates to the elementary rate constant of the O* + H* reaction, *k*_1_, as shown in eq 9. We can thus determine *k*_1_ from the slope of a linear fit of *k*_eff_ plotted against [H*] or [D*] while keeping the intercept fixed at zero, see also the dashed lines in Figure 1(b).

9 $$k_{\mathrm{eff}}=\frac{1}{2}k_{1}\left[H^{*}\right]$$

Figure 3(a) shows *k*_1_ as a function of surface temperature for reactions involving hydrogen and deuterium as black and red squares, respectively. The solid lines are Arrhenius fits; fit results are given in Table 1.

**Figure 3:**
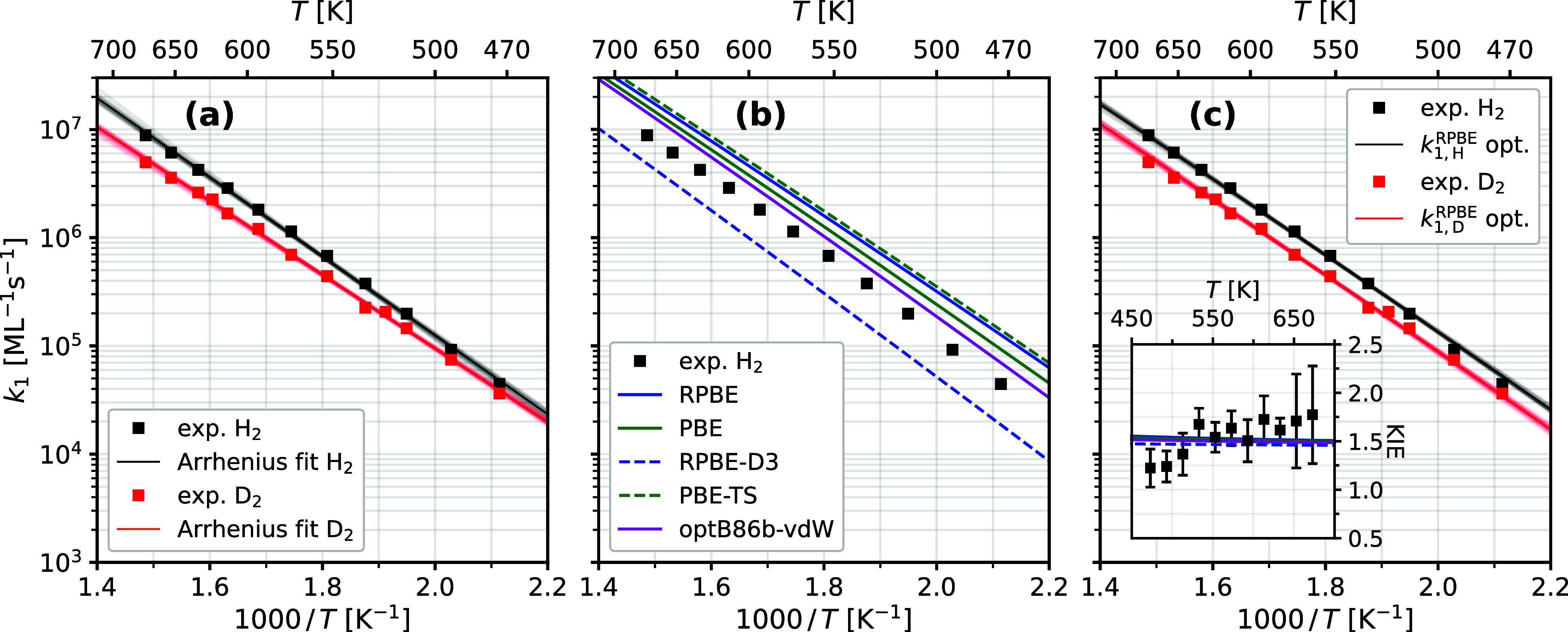
Second-order rate constants for the rate-determining step of hydrogen oxidation on Pt(332). (a) Experimental data (squares) and Arrhenius fits (solid lines) for O* + H* → OH* (black) and O* + D* → OD* (red). Transparent regions around the solid lines indicate 2σ-uncertainty intervals of the fit. (b) Rate constants from experiment (squares) and from a transition state theory (TST) model (lines) based on DFT data obtained with five different exchange correlation functionals as indicated in the legend. (c) DFT-TST rate constants for the RPBE functional multiplied by 0.52 exp­(−9 meV/*k*_B_ *T*) (lines) for optimized agreement with the experiment (squares). The inset shows the kinetic isotope effect, KIE = *k*_1,H_/*k*_1,D_, calculated from the experiment (squares) and predicted by the DFT-TST model (lines) with all five functionals that were shown in panel (b). Note that the lines largely overlap, for numerical values see Table 3.

**Table 1:** Experimental Arrhenius Activation Energies *E*_A_ and Prefactors *A* for the O* + H* → OH* and O* + D* → OD* reaction on Pt(332)^a^

	*E*_A_ [eV]	*A* [ML^–1^s^–1^]
O* + H* → OH*	0.725 ± 0.029	10^12.40±0.26^
O* + D* → OD*	0.677 ± 0.025	10^11.80±0.24^

a Error bars indicate 95% confidence.

We performed additional DFT calculations for TST predictions of *k*_1_ using five commonly employed GGA exchange-correlation functionals: RPBE, PBE, RPBE-D3, PBE-TS and optB86b-vdW. The results are shown in Figure 3(b) for the light hydrogen isotope along with the experimentally derived rate constants. An equivalent figure for the deuterium isotope is shown in SI Figure S4. Notably, theory and experiment agree within a factor of 2.5 independent of choice of functional, see also Table 2.

**Table 2:** Comparison of Experimental and Theoretical Rate Constants for the O* + H*­(D*) Reaction on Pt(332)^a^

	*k*_exp._/*k*_DFT_	Δ*E* [eV]	*a*
RPBE	0.43	+0.009 ± 0.020	0.52 ± 0.22
PBE	0.54	–0.011 ± 0.020	0.43 ± 0.18
RPBE-D3	2.14	–0.063 ± 0.020	0.58 ± 0.24
PBE-TS	0.39	+0.010 ± 0.020	0.47 ± 0.20
optB86b-vdW	0.65	–0.032 ± 0.020	0.34 ± 0.14

a The first column shows the ratio of experimental and theoretical rate constants averaged over the experimental temperature range for both isotopes. In the next two columns we separate deviations it into errors Δ*E* in the calculated DFT barrier heights, and errors *a* in modelling of the prefactor. Error bars indicate 95% confidence.

In order to separately evaluate errors in DFT-calculated reaction barrier heights Δ*E* versus errors in prefactor modeling *a*, we apply a multiplicative correction term of the form *a* × exp­(−Δ*E*/*k*_B_ *T*) to each DFT-TST predicted *k*_1_, using *a* and Δ*E* as fit parameters in order to match experimentally derived rate constants. For each choice of functional the same parameters are used for both isotopes, and the optimization procedure is performed globally for the O* + H* and O* + D* data sets. This ensures that all isotope effects are accounted for within the rate model. Figure 3(c) shows the results of such an error evaluation procedure and the resulting fit for both isotopes when using the RPBE functional. Table 2 summarizes the results of the error analysis in terms of the fitted parameters *a* and Δ*E* for all five functionals.

The prefactor corrections *a* range between 1/3 and 1/2, values consistent with typical transition state recrossing corrections,^46–52^ which were not included in our rate model. By adding Δ*E* from Table 2 to each DFT-derived barrier height *E*_0_ listed in Table 3, we obtain an approximately functional independent estimate of the true zero-point energy corrected barrier height for both isotopic variants of reaction 3. This is shown in the bottom row of Table 3 and is labeled “experiment”. The error bars account for both experimental uncertainties and residual variation between the functionals.

**Table 3:** ZPE-Corrected Reaction Barrier Heights *E*_0_ and Kinetic Isotope Effects (KIE) for the O* + H*­(D*) Reaction on Pt(332)^a^

	*E*_0_ (H)	*E*_0_ (D)	KIE
RPBE	0.749	0.756	1.53
PBE	0.768	0.775	1.51
RPBE-D3	0.834	0.842	1.46
PBE-TS	0.749	0.755	1.51
optB86b-vdW	0.784	0.790	1.50
experiment	0.760 ± 0.030	0.766 ± 0.030	1.55 ± 0.27

a DFT results are shown using five different GGA exchange-correlation functionals. The last column shows the temperature averaged kinetic isotope effect, defined as KIE = *k*_1,H_/*k*_1,D_. Experimental error bars indicate 95% confidence.

The inset of Figure 3(c) shows the DFT-TST predicted kinetic isotope effect, KIE = *k*_1,H_/*k*_1,D_. Temperature averaged values of the KIE are also reported in Table 3. The theoretically predicted KIE is also in excellent agreement with observation and depends only very weakly on the choice of functional.

## 4 Discussion

In this work, we have presented HRR-VRK data of the hydrogen oxidation reaction on a Pt(332) single crystal surface under low adsorbate coverage conditions (<6 × 10^–3^ ML) with a pulse of O_2_ initiating the reaction. We identified the reaction O* + H* → OH* taking place at B-type step sites as rate limiting for which we extracted second order rate constants that serve as an experimental benchmark of a TST rate model based on DFT-GGA calculations. We evaluated the performance of 5 commonly used functionals and found the predicted rate constants to be in remarkably good agreement with experimentally derived rate constants. We are able to evaluate the error in the predicted reaction barrier height associated with each choice of functional, which we find to be remarkably small; the mean absolute error is 25 meV.

In the following, we first discuss our assignment of the RDS in the context of previous hydrogen oxidation studies and then comment on the agreement of DFT computed barrier heights and TST-derived rates with the experiment.

### 4.1 A Note about the Mechanism and Previous Studies

The hydrogen oxidation reaction on platinum surfaces has been extensively studied in the past and many apparently conflicting proposals have been made concerning the reaction mechanism. This lack of consensus results from the fact that the mechanism depends strongly on reaction conditions. At low temperatures (<170 K) where water may condense to the surface and actively participates in the reaction, autocatalytic behavior has been observed.^53,54^ At higher temperatures where water desorbs rapidly, most studies agree on a sequential water formation mechanism via reactions 3 to 5;^43,44,55,56^ however, there is no consensus on the RDS and there is a wide range of reported activation energies (0.2 to 0.8 eV).^40,41,43,44,56–58^ MBRS experiments indicated that defects enhance reactivity and that defect concentrations as small as 10^–5^ can dominate the observed kinetics.^57^ The mechanism also has been shown to depend on experimental parameters, particularly the relative fluxes of hydrogen and oxygen to the surface.^43^ A previous VRK study of hydrogen oxidation on Pt(111) under conditions of high oxygen coverage (∼0.1 ML) with a molecular beam of H_2_ initiating the reaction identified second order kinetics, strongly suggesting that reaction 5 is rate limiting under those conditions.^42^ One MBRS study was performed at relatively low oxygen coverage (≤0.04 ML).^56^ Although the coverages were more than an order of magnitude higher than in the present work, the reported rate constants are similar. The authors, who had no access to theoretical tools concluded incorrectly that reaction 4 is the RDS based on the shape of the MBRS curves.^56^

This study goes beyond past work in that it exploits the high sensitivity of HRR-VRK together with ab initio kinetics methods to clarify the reaction mechanism at extraordinarily low oxygen and hydrogen coverages. By restricting conditions to low coverage and using a controlled density of well-defined steps, we have been able to obtain data that can be accurately modeled by a first-principles rate theory.

### 4.2 Discussion of the Agreement between DFT-GGA and Experiment

We computed rate constants for the O* + H*­(D*) → OH*­(OD*) reaction based on DFT-derived barrier heights and partition functions and found that the results are only weakly sensitive to the choice of the DFT-GGA functional and to van der Waals corrections. All five functionals studied here gave rate constants within a factor of 2.5 of experiment. Since high precision kinetic data was obtained over a wide temperature range, we were able to distinguish barrier height and prefactor contributions to the deviations between TST and experimentally derived rate constants.

The multiplicative prefactor corrections required to obtain agreement with the experimental rate constants are approximately 0.5 for all functionals. In principle, these deviations from unity could arise from tunneling, transition state recrossing, or anharmonic contributions to the partition functions. However, significant tunneling contributions can be excluded, because the experimental KIE is well reproduced by the rate model which neglects tunneling. Although the reaction coordinate involves significant hydrogen atom motion, see SI Figure S3, tunneling appears to be negligible due to the elevated temperatures of the experiment. Tunneling corrections calculated using a simplified one-dimensional Eckart model indicate that tunneling would only become significant at temperatures lower than those studied here. We believe that neglect of transition state recrossing is likely to be the main source of the prefactor discrepancy; since, reported recrossing corrections are similar in magnitude to the deviations seen here. For example, they range from 0.3 to 0.5 for high temperature hydrogen abstraction reactions in the gas phase,^50,52^ from 0.7 to 0.9 for surface diffusion,^51^ and from 0.4 to 0.9 for dissociative adsorption and recombinative desorption of H_2_ on Cu(111), Ag(111), and Pt(111).^46–49^

We note that our rate model explicitly accounts for anharmonic and quantum mechanical effects for the H* atom, thus any neglected anharmonic contributions originate from the O* atom or from the [O···H]* transition state. We expect these effects to be small for two reasons. First, previous work has shown that employing the modified harmonic oscillator partition function, eq 2, yields results within a factor of 1.5 compared to more reliable methods for computing adsorbate partition functions.^38^ Second, the anharmonic influence on the O* atom and [O···H]* transition state partition functions are likely to be similar and since they appear in eq 1 as a ratio, their effect on the rate constant will tend to cancel. Similarly, excluding the metal’s degrees of freedom when calculating partition functions does affect the absolute values of the initial state and transition state partition functions, but the changes are approximately equal multiplicative factors and thus largely cancel out when computing the TST rate constant. As shown in Section S4.5 of the SI, calculating partition functions only for the adsorbates on a frozen metal surface results in relative errors in the rate constant of less than 1.5% throughout the temperature range studied here.

The errors of DFT-computed barrier heights for the five functionals studied here are all within 0.063 eV of the experimental benchmark, with a mean absolute error of 0.025 eV. Results using one functional (RPBE-D3) appear as an outlier, deviating by more than four times the mean absolute error of the other four results and being the only calculation that predicts a rate constant smaller than the benchmark values. This suggests that RPBE-D3 likely overestimates the importance of van der Waals interactions—two other functionals designed to account for van der Waals interactions give results insignificantly different than those obtained from functionals neglecting van der Waals interactions.

The barrier height errors found here for the five tested functionals, are much lower than the errors found by previous benchmarks of DFT applied to gas-surface reactions. For example, Wellendorf et al. reported DFT chemisorption energies for 25 different molecule–surface systems obtained with five different commonly used GGA functionals, showing mean absolute errors on the order of 0.4 eV compared to the experiment.^20^

We attribute the large errors seen for gas-surface reactions to the difficulty of DFT-GGA functionals in describing both molecular systems and extended systems, such as solids and surfaces, at the same time.^21–23^ Functionals, constructed to describe the properties of gas phase molecules, often perform poorly for solids and vice versa.^21–25^ As a result, adsorption energies—defined as the energy difference between a molecule in the gas phase and adsorbed on a surface—are particularly sensitive to these difficulties and often deviate from experimental values.

This problem has been recognized in other fields, such as computational electrocatalysis, where uncertainties in DFT models have been shown to largely originate from errors in the calculation of gas phase energies.^59–61^ To address this, semiempirical corrections for the energies of gas phase molecules have been proposed,^61^ substantially improving the agreement between computational models and the experiment. Similar developments in the field of surface science have been made, where semiempirically weighted mixtures of two GGA functionals were able to successfully predict a range of dissociative chemisorption barriers.^12,13,62^

## 5 Conclusions

A central result of this study is the establishment of an accurate benchmark for the rate constant and the barrier height of an elementary on-surface reaction. Comparing this to barrier heights calculated with DFT, we find errors that are much smaller than would be anticipated from benchmarks of gas-surface reactions. The importance of achieving accurate barrier heights can be better appreciated by considering the effect these errors have on predicted rates of chemical reactions. At 600 K—a temperature relevant to our work—a 0.4 eV barrier height error, typical of gas-surface reactions, leads to deviations in predicted reaction rates by a factor of 2300, whereas the 0.025 eV error found in this work results in a much smaller deviation of only a factor of 1.6.

It is interesting to speculate that small errors will also apply to other on-surface reactions, as they avoid the need for an accurate description of both gas phase and adsorbed molecules by the same method, which is known to be difficult for DFT at the GGA level.^21^ If this speculation holds, accurate first-principles simulations of processes that involve on-surface reactions could be obtained by correcting only the gas phase energies involved, thus providing a powerful tool for understanding catalytic systems. We hope this work will stimulate further benchmarking of DFT for reactions on surfaces to test the generality of our findings.

## Supplementary Material



## Notes

Hydrogen surface coverage determination, partition function and TST rate constant calculations, microkinetic model construction, degree of rate control analysis (PDF)

## References

[1] Linic S., Barteau M. A. (2003). Control of Ethylene
Epoxidation Selectivity by Surface
Oxametallacycles. J. Am. Chem. Soc..

[2] Grabow L. C., Mavrikakis M. (2011). Mechanism
of methanol synthesis on Cu through CO_2_ and CO hydrogenation. ACS Catal..

[3] Rommens K. T., Saeys M. (2023). Molecular Views on Fischer–Tropsch Synthesis. Chem. Rev..

[4] Becke A. D. (1988). Density-functional
exchange-energy approximation with correct asymptotic behavior. Phys. Rev. A.

[5] Perdew J. P., Burke K., Ernzerhof M. (1996). Generalized
Gradient Approximation
Made Simple. Phys. Rev. Lett..

[6] Perdew J. P., Chevary J. A., Vosko S. H. (1992). Atoms, molecules, solids,
and surfaces: Applications of the generalized gradient approximation
for exchange and correlation. Phys. Rev. B.

[7] Guo M., Ji M., Cui W. (2022). Theoretical
investigation of HER/OER/ORR catalytic
activity of single atom-decorated graphyne by DFT and comparative
DOS analyses. Appl. Surf. Sci..

[8] Wu P., Du P., Zhang H., Cai C. (2012). Graphyne As a Promising Metal-Free
Electrocatalyst for Oxygen Reduction Reactions in Acidic Fuel Cells:
A DFT Study. J. Phys. Chem. C.

[9] Chen Z. W., Chen L. X., Wen Z., Jiang Q. (2019). Understanding electro-catalysis
by using density functional theory. Phys. Chem.
Chem. Phys..

[10] Reuter K., Scheffler M. (2003). Composition
and structure of the RuO_2_(110)
surface in an O_2_ and CO environment: Implications for the
catalytic formation of CO_2_. Phys.
Rev. B.

[11] Honkala K., Hellman A., Remediakis I. N. (2005). Ammonia Synthesis from
First-Principles Calculations. Science.

[12] Díaz C., Pijper E., Olsen R. A. (2009). Chemically
Accurate
Simulation of a Prototypical Surface Reaction: H_2_ Dissociation
on Cu(111). Science.

[13] Tchakoua T., Gerrits N., Smeets E. W. F., Kroes G. J. (2023). SBH17: Benchmark
Database of Barrier Heights for Dissociative Chemisorption on Transition
Metal Surfaces. J. Chem. Theory Comput..

[14] van
Bree R. A. B., Gerrits N., Kroes G.-J. (2024). Dissociative chemisorption
of O_2_ on Al(111): dynamics on a potential energy surface
computed with a non-self-consistent screened hybrid density functional
approach. Faraday Discuss..

[15] van
Bree R. A. B., Kroes G. J. (2025). Limits of BOSS DFT: O_2_ + Al(111) Dynamics on a Screened Hybrid Van der Waals DFT Potential
Energy Surface. J. Phys. Chem. C.

[16] Nattino F., Ueta H., Chadwick H. (2014). Ab Initio Molecular
Dynamics Calculations versus Quantum-State-Resolved Experiments on
CHD_3_ + Pt(111): New Insights into a Prototypical Gas–Surface
Reaction. J. Phys. Chem. Lett..

[17] Campbell C. T. (2019). Energies
of Adsorbed Catalytic Intermediates on Transition Metal Surfaces:
Calorimetric Measurements and Benchmarks for Theory. Acc. Chem. Res..

[18] Hensley A. J. R., Ghale K., Rieg C. (2017). DFT-Based
Method for
More Accurate Adsorption Energies: An Adaptive Sum of Energies from
RPBE and vdW Density Functionals. J. Phys. Chem.
C.

[19] Campbell C. T., Fingerhut J., Wodtke A. M. (2025). Experimental energies of formation
reactions for adsorbates on late transition metal surfaces: A database
update. Surf. Sci..

[20] Wellendorff J., Silbaugh T. L., Garcia-Pintos D. (2015). A benchmark database
for adsorption bond energies to transition metal surfaces and comparison
to selected DFT functionals. Surf. Sci..

[21] Kurth S., Perdew J. P., Blaha P. (1999). Molecular
and solid-state tests of
density functional approximations: LSD, GGAs, and meta-GGAs. Int. J. Quantum Chem..

[22] Haas P., Tran F., Blaha P., Schwarz K. (2011). Construction of an
optimal GGA functional for molecules and solids. Phys. Rev. B.

[23] Peverati R., Truhlar D. G. (2012). Exchange–Correlation Functional with Good Accuracy
for Both Structural and Energetic Properties while Depending Only
on the Density and Its Gradient. J. Chem. Theory
Comput..

[24] Csonka G.
I., Perdew J. P., Ruzsinszky A. (2009). Assessing the performance
of recent density functionals for bulk solids. Phys. Rev. B.

[25] Perdew J. P., Burke K., Ernzerhof M. (1998). Perdew, Burke, and Ernzerhof Reply. Phys. Rev. Lett..

[26] Harding D. J., Neugebohren J., Hahn H. (2017). Ion and
velocity map
imaging for surface dynamics and kinetics. J.
Chem. Phys..

[27] Nitz F., Hörandl S., Golibrzuch K. (2025). Multi-mass velocity-resolved
kinetics of surface reactions at 100 kHz acquisition rate. Rev. Sci. Instrum..

[28] Kresse G., Furthmüller J. (1996). Efficiency of ab-initio total energy calculations for
metals and semiconductors using a plane-wave basis set. Comput. Mater. Sci..

[29] Kresse G., Furthmüller J. (1996). Efficient iterative schemes for ab
initio total-energy
calculations using a plane-wave basis set. Phys.
Rev. B.

[30] Kresse G., Hafner J. (1993). Ab initio molecular
dynamics for liquid metals. Phys. Rev. B.

[31] Methfessel M., Paxton A. T. (1989). High-precision sampling
for Brillouin-zone integration
in metals. Phys. Rev. B.

[32] Monkhorst H. J., Pack J. D. (1976). Special points for
Brillouin-zone integrations. Phys. Rev. B.

[33] Hammer B., Hansen L. B., Nørskov J. K. (1999). Improved
adsorption energetics within
density-functional theory using revised Perdew-Burke-Ernzerhof functionals. Phys. Rev. B.

[34] Grimme S., Antony J., Ehrlich S., Krieg H. (2010). A consistent
and accurate
ab initio parametrization of density functional dispersion correction
(DFT-D) for the 94 elements H-Pu. J. Chem. Phys..

[35] Tkatchenko A., Scheffler M. (2009). Accurate Molecular Van Der Waals Interactions from
Ground-State Electron Density and Free-Atom Reference Data. Phys. Rev. Lett..

[36] Klimeš J., Bowler D. R., Michaelides A. (2011). Van der Waals
density functionals
applied to solids. Phys. Rev. B.

[37] Henkelman G., Uberuaga B. P., Jónsson H. (2000). A climbing
image nudged elastic band
method for finding saddle points and minimum energy paths. J. Chem. Phys..

[38] Blöndal K., Sargsyan K., Bross D. H., Ruscic B., Goldsmith C. F. (2023). Configuration
Space Integration for Adsorbate Partition Functions: The Effect of
Anharmonicity on the Thermophysical Properties of CO–Pt(111)
and CH_3_OH–Cu­(111). ACS Catal..

[39] Borodin D., Hertl N., Park G. B. (2022). Quantum effects in thermal
reaction rates at metal surfaces. Science.

[40] Gdowski G. E., Madix R. J. (1982). The kinetics and mechanism of the
hydrogen-oxygen reaction
on Pt­(S)–[9(111) × (100)]. Surf.
Sci..

[41] Ljungström S., Kasemo B., Rosen A., Wahnström T., Fridell E. (1989). An experimental study of the kinetics of OH and H_2_O formation on Pt in the H_2_ + O_2_ reaction. Surf. Sci..

[42] Borodin D., Schwarzer M., Hahn H. W. (2021). The
puzzle of rapid
hydrogen oxidation on Pt(111). Mol. Phys..

[43] Anton A. B., Cadogan D. C. (1991). Kinetics of water
formation on Pt(111). J. Vacuum Sci. Technol.
A.

[44] Hellsing B., Kasemo B., Zhdanov V. P. (1991). Kinetics
of the hydrogen-oxygen reaction
on platinum. J. Catal..

[45] Evans M. G., Polanyi M. (1938). Inertia and driving
force of chemical reactions. Trans. Faraday
Soc..

[46] Galparsoro O., Kaufmann S., Auerbach D. J., Kandratsenka A., Wodtke A. M. (2020). First principles rates for surface chemistry employing
exact transition state theory: application to recombinative desorption
of hydrogen from Cu(111). Phys. Chem. Chem.
Phys..

[47] Zhang L., Nitz F., Borodin D., Wodtke A. M., Guo H. (2025). Ring Polymer
Molecular Dynamics Rates for Hydrogen Recombinative Desorption on
Pt(111). Precis. Chem..

[48] Nitz F., Zhang L., Hertl N. (2024). Thermal Rates and High-Temperature
Tunneling from Surface Reaction Dynamics and First-Principles. J. Am. Chem. Soc..

[49] Zhang L., Zuo J., Suleimanov Y. V., Guo H. (2023). Ring Polymer Molecular
Dynamics Approach to Quantum Dissociative Chemisorption Rates. J. Phys. Chem. Lett..

[50] Suleimanov Y. V., Espinosa-Garcia J. (2016). Recrossing and Tunneling in the Kinetics
Study of the
OH + CH_4_ → H_2_O + CH_3_ Reaction. J. Phys. Chem. B.

[51] Sharia O., Henkelman G. (2016). Analytic dynamical corrections to
transition state
theory. New J. Phys..

[52] Gonzalez-Lavado E., Corchado J. C., Suleimanov Y. V., Green W. H., Espinosa-Garcia J. (2014). Theoretical
Kinetics Study of the O­(^3^P) + CH_4_/CD_4_ Hydrogen Abstraction Reaction: The Role of Anharmonicity, Recrossing
Effects, and Quantum Mechanical Tunneling. J.
Phys. Chem. A.

[53] Sachs C., Hildebrand M., Völkening S., Wintterlin J., Ertl G. (2002). Reaction fronts in the oxidation of hydrogen on Pt(111): Scanning
tunneling microscopy experiments and reaction–diffusion modeling. J. Chem. Phys..

[54] Völkening S., Bedürftig K., Jacobi K., Wintterlin J., Ertl G. (1999). Dual-Path Mechanism for Catalytic Oxidation of Hydrogen on Platinum
Surfaces. Phys. Rev. Lett..

[55] Gland J. L., Fisher G. B., Kollin E. B. (1982). The hydrogen-oxygen
reaction over
the Pt(111) surface: Transient titration of adsorbed oxygen with hydrogen. J. Catal..

[56] Anton A. B., Cadogan D. C. (1990). The mechanism and kinetics of water
formation on Pt(111). Surf. Sci..

[57] Verheij L. K., Hugenschmidt M. B. (1998). On the
mechanism of the hydrogen–oxygen reaction
on Pt(111). Surf. Sci..

[58] Eisert F., Rosén A. (1996). In situ investigation
of the catalytic reaction H_2_ + 1/2 O_2_ →
H_2_O on Pt(111) with
second-harmonic generation. Phys. Rev. B.

[59] Almeida M. O., Kolb M. J., Lanza M. R. V., Illas F., Calle-Vallejo F. (2022). Gas-Phase
Errors Affect DFT-Based Electrocatalysis Models of Oxygen Reduction
to Hydrogen Peroxide. ChemElectroChem.

[60] Sargeant E., Illas F., Rodríguez P., Calle-Vallejo F. (2021). Importance
of the gas-phase error correction for O_2_ when using DFT
to model the oxygen reduction and evolution reactions. J. Electroanal. Chem..

[61] Granda-Marulanda L. P., Rendón-Calle A., Builes S. (2020). A Semiempirical Method
to Detect and Correct DFT-Based Gas-Phase Errors and Its Application
in Electrocatalysis. ACS Catal..

[62] Kroes G.-J. (2015). Toward
a Database of Chemically Accurate Barrier Heights for Reactions of
Molecules with Metal Surfaces. J. Phys. Chem.
Lett..

