# Supplementary material for: An Experimental Benchmark for the Barrier Height of an On-Surface Reaction: Hydrogen Oxidation on Pt(332)
Source: J Am Chem Soc. 2025 Oct 3;147(41):37658–66. doi: 10.1021/jacs.5c12862 (PMC12532188; doi:10.1021/jacs.5c12862)
Supplement: Supplementary file 1 [file ja5c12862_si_001.pdf]

Supporting Information for

An experimental benchmark for the barrier height of an on-surface reaction: Hydrogen oxidation on Pt(332)

Florian Nitz[1,2], Stefan Hörandl[1,2], Michael Schwarzer[2], Theofanis Kitsopoulos[3], Daniel J. Auerbach[2], Alec M. Wodtke[1,2,4]*

[1] Institute for Physical Chemistry, Georg-August University of Goettingen, Tammannstraße 6, 37077 Goettingen, Germany.

[2] Department of Dynamics at Surfaces, Max Planck Institute for Multidisciplinary Sciences, Am Fassberg 11, 37077 Goettingen, Germany.

[3] School of Mathematics and Natural Sciences, University of Southern Mississippi, Hattiesburg, Mississippi 39406, USA.

[4] International Center for Advanced Studies of Energy Conversion, Georg-August University of Goettingen, Tammannstraße 6, 37077 Goettingen, Germany.

Email: *alec.wodtke@mpinat.mpg.de

Contents

S1. Hydrogen Coverage Determination 3

S1.1. Thermal Sticking Coefficient of Hydrogen 4

S2. Optimized Lattice Constants using Density Functional Theory 6

S3. Experimental Rate Constants for Hydrogen (H_2) Oxidation 6

S4. Transition State Theory Rate Constants for O*+H*→OH* 7

S4.1. O*+H*→OH* Barrier Height 7

S4.2. Oxygen Atom Partition Function 8

S4.3. [O···H]* Transition State Partition Function 9

S4.4. Comparison to Experimental Rate Constants 10

S4.5. Effect of the Static Surface Approximation in Partition Function Calculations 11

S5. Transition State Theory Rate Constants for OH*+H*→H_2O* and 2OH*→H_2O*+O* 12

S5.1. OH*+H*→H_2O* and 2OH*→H_2O*+O* Barrier Heights 12

S5.2. OH* Partition Function 14

S5.3. [OH···H]* and [OH···OH]* Transition State Partition Function 14

S6. Microkinetic Model of Hydrogen Oxidation on Pt(332) 16

S6.1. Construction of the Microkinetic Model 16

S6.2. Degree of Rate Control Analysis 17

S7. Additional References 18

S1. Hydrogen Coverage Determination

The hydrogen oxidation experiments described in the main text were initiated by a molecular beam of pure oxygen impinging onto Pt(332) surface pre-covered with hydrogen atoms at a known surface coverage θ, established by introducing H_2 gas into the ultrahigh vacuum (UHV) chamber using a leak valve. In the following, we describe the determination of θ from the ion gauge pressure p_{IG} reading.

First, we correct p_{IG} for the influence of the partial pressure of other gases in the UHV chamber. The true H_2 partial pressure $p_{\mathrm{H_2}}$ during the experiment is calculated using the base pressure p_{IG}^0 of all gases in the UHV chamber with the leak valve closed, the fraction $f_{\mathrm{H_2}}$ of H_2 in the base pressure and the sensitivity factor S_{IG} of the ion gauge for H_2.

$$p_{\mathrm{H_2}} = \left(p_{\mathrm{IG}} - p_{\mathrm{IG}}^0(1 - f_{\mathrm{H_2}})\right) \times S_{\mathrm{IG}} \qquad \text{(S1)}$$

We use S_{IG} as specified by the manufacturer and determine $f_{\mathrm{H_2}}$ using a quadrupole mass spectrometer with an electron bombardment ionizer running at an electron energy similar to that of the ion gauge. Note that during the experiment, hydrogen is the predominant gas in the UHV chamber, and the hydrogen partial pressure can be accurately approximated by the total pressure in the chamber.

The relationship between $p_{\mathrm{H_2}}$ and θ is given by a Langmuir isotherm. A steady state between the hydrogen adsorption rate r_{ads} from the gas phase and the recombinative hydrogen desorption rate r_{des} defines the hydrogen atom coverage θ. The adsorption rate from the gas phase is given by equation (S2).

$$r_{\mathrm{ads}} = 2\,\langle S_0\rangle(T_{\mathrm{g}})\left(1 - \frac{\theta}{\theta_{\max}}\right)^2 \frac{p_{\mathrm{H_2}}}{\sqrt{2\pi m k_{\mathrm{B}} T_{\mathrm{g}}}}\, N_{\mathrm{sites}}^{-1} \qquad \text{(S2)}$$

$\langle S_0\rangle(T_{\mathrm{g}})$ is the incidence angle averaged thermal sticking coefficient of hydrogen at ambient gas temperature $T_{\mathrm{g}} = 298$ K, determined in section S1.1, m is the mass of the H_2 molecule, and $\theta_{\max}$ is the saturation coverage of hydrogen atoms which is assumed to be $\theta_{\max} = 1$ ML. Note that the exact value of $\theta_{\max}$ does not influence the results presented here since $\theta \ll \theta_{\max}$ for all conditions of this work. N_{sites} is the number of adsorption sites per cm^2 of the Pt surface. We calculated N_{sites} from the experimental lattice constant (3.923 Å [1]) and obtained $N_{\mathrm{sites}} = 1.5 \cdot 10^{15}\ \mathrm{cm}^{-2}$.

The desorption rate r_{des} depends on the recombinative desorption rate constant $k_{\mathrm{rec}}(T_{\mathrm{s}})$ at the surface temperature T_{s}, see equation (S3). Accurate values for $k_{\mathrm{rec}}(T_{\mathrm{s}})$ from Pt(332) have been determined previously [2] using velocity-resolved kinetics (VRK) experiments and are used here. That study also introduced a physical model capable of reliably predicting experimental desorption rate constants [2], which we employ here to estimate $k_{\mathrm{rec}}(T_{\mathrm{s}})$ at surface temperatures for which no experimental rate constants are available.

$$r_{\mathrm{des}} = 2\, k_{\mathrm{rec}}(T_{\mathrm{s}})\, \theta^2\, N_{\mathrm{sites}} \qquad \text{(S3)}$$

Equation (S3) assumes that the hydrogen surface coverage is not affected by the reaction with oxygen and at all times determined by the adsorption-desorption equilibrium alone. In a steady state, adsorption and desorption rate are equal, $r_{\mathrm{ads}} = r_{\mathrm{des}}$. By equating (S2) and (S3), we obtain equation (S4), and after rearrangement equation (S5).

$$2\,\langle S_0\rangle(T_\mathrm{g})\left(1-\frac{\theta}{\theta_\mathrm{max}}\right)^2\;\frac{p_{\mathrm{H}_2}}{\sqrt{2\pi m k_\mathrm{B} T_\mathrm{g}}}\;N_\mathrm{sites}^{-1} = 2\,k_\mathrm{rec}(T_\mathrm{s})\,\theta^2\,N_\mathrm{sites} \tag{S4}$$

$$\frac{\theta^2}{\left(1-\frac{\theta}{\theta_\mathrm{max}}\right)^2} = \frac{\langle S_0\rangle(T_\mathrm{g})}{k_\mathrm{rec}(T_\mathrm{s})}\;\frac{p_{\mathrm{H}_2}}{\sqrt{2\pi m k_\mathrm{B} T_\mathrm{g}}}\;N_\mathrm{sites}^{-2} \equiv K\left(p_{\mathrm{H}_2},T_\mathrm{s},T_\mathrm{g}\right) \tag{S5}$$

All terms on the right side of equation (S5) will be abbreviated with $K\left(p_{\mathrm{H}_2},T_\mathrm{s},T_\mathrm{g}\right)$ in the following. The hydrogen coverage θ can then be obtained from:

$$\theta = \frac{\sqrt{K\left(p_{\mathrm{H}_2},T_\mathrm{s},T_\mathrm{g}\right)}\,\theta_\mathrm{max}}{\sqrt{K\left(p_{\mathrm{H}_2},T_\mathrm{s},T_\mathrm{g}\right)}+\theta_\mathrm{max}}. \tag{S6}$$

For the deuterium oxidation experiments, the same procedure is applied to calculate the deuterium surface coverage, while accounting for changes in the ion gauge sensitivity factor S_IG, the higher mass of the deuterium molecule in equation (S4) and the isotope effect in the recombinative desorption rate constants.

For the convenience of future workers who may need access to the quantitative Langmuir isotherm we provide an empirical formula based on our work that can be used to calculate $K\left(p_{\mathrm{H}_2},T_\mathrm{s},T_\mathrm{g}\right)$ for hydrogen:

$$K\left(p_{\mathrm{H}_2},T_\mathrm{s},T_\mathrm{g}\right) = 1.22\times10^{-7}\times\frac{p_{\mathrm{H}_2}}{\mathrm{mbar}}\times10^{\left[1.918\left(\frac{298\,\mathrm{K}}{T_\mathrm{s}}\right)^2+12.21\left(\frac{298\,\mathrm{K}}{T_\mathrm{s}}\right)\right]}\times\left(\frac{298\,\mathrm{K}}{T_\mathrm{g}}\right)^{0.5}, \tag{S7}$$

and for deuterium:

$$K\left(p_{\mathrm{D}_2},T_\mathrm{s},T_\mathrm{g}\right) = 1.57\times10^{-7}\times\frac{p_{\mathrm{D}_2}}{\mathrm{mbar}}\times10^{\left[2.295\left(\frac{298\,\mathrm{K}}{T_\mathrm{s}}\right)^2+11.44\left(\frac{298\,\mathrm{K}}{T_\mathrm{s}}\right)\right]}\times\left(\frac{298\,\mathrm{K}}{T_\mathrm{g}}\right)^{0.5}. \tag{S8}$$

S1.1. Thermal Sticking Coefficient of Hydrogen

Equations (S5) and (S6) indicate that accurately determining the steady state hydrogen coverage requires knowledge of the incidence angle averaged thermal sticking coefficient $\langle S_0\rangle$ for H_2 at ambient gas temperature T_g. To compute $\langle S_0\rangle$, we first determine the kinetic energy dependent initial sticking coefficient, $S_0(E)$, and then perform an average over all kinetic energies and incidence angles.

The velocity-resolved kinetics (VRK) method can be used to measure the velocity or kinetic energy distributions of desorbing molecules, see refs. 3,4 for details. Here, we made use of this capability and measured the energy-resolved thermal desorption flux of HD molecules produced when a mixed H_2/D_2 molecular beam impinges on a clean Pt(332) surface 523 K. We verified that the incident beam contained no HD, ensuring background-free thermal desorption conditions. We use this data and the principle of detailed balance to calculate the sticking coefficient as a function of kinetic energy; the results are shown as black crosses in Figure S1. Here, the kinetic energy E corresponds to the normal kinetic energy of product HD molecules, as the flux is probed using an ionization laser positioned at the surface normal.

The VRK experiment provides the energy dependence of $S_0(E)$, however, the data is obtained on an arbitrary scale. To place the results on an absolute scale, we scaled the VRK data for agreement with previously reported King and Wells sticking probability measurements of D_2 on a curved Pt crystal [5]. We used King and Wells data for a B-type step density of 17%, shown as black squares in Figure S1. This reference is sufficiently similar to the Pt(332) surface used in our experiment, which has 16.7% B-type steps. Additionally, we assume no isotopic differences in $S_0(E)$, an assumption supported by prior studies on Pt(111) [6] and Pt(533) [7].

Next, we fit the scaled VRK data to obtain a continuous kinetic energy dependence of the initial sticking probability, which is necessary for the thermal averaging procedure. Previous studies have shown that $S_0(E)$ on stepped platinum surfaces can be well described by the functional form given in equation (S9) [5,8], where the parameters A, α, B and C are optimized for agreement with experimental data.

$$S_0(E) = A\exp(-\alpha E) + B + CE \qquad \text{(S9)}$$

We determined the parameters $A = 0.44$, $\alpha = 86\ \text{eV}^{-1}$, $B = 0.12$ and $C = 1.18\ \text{eV}^{-1}$ by fitting equation (S9) to the scaled VRK data. The model for $S_0(E)$ using optimized parameters is shown as the red line in Figure S1.

The incidence angle averaged thermal sticking coefficient $\langle S_0 \rangle$ is calculated numerically following the procedure described in ref. 9, assuming normal energy scaling—consistent with previous reports [6]—and cylindrical symmetry. While the assumption of cylindrical symmetry is not strictly valid for the stepped Pt(332) surface, any anisotropies are expected to largely cancel out due to the integration over all angles, see also SI section 5c in ref. 2. The calculation yields a value of $\langle S_0 \rangle(T = 298\ \text{K}) = 0.29 \pm 0.01$.

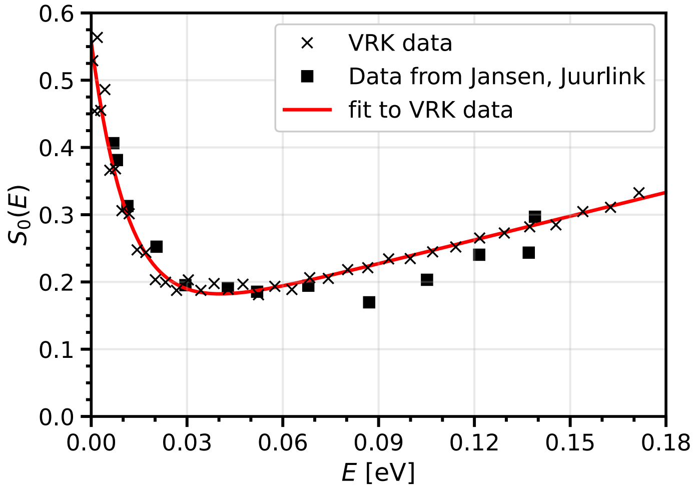

Figure S1: Initial sticking probability $S_0(E)$ of hydrogen on Pt(332). E is the kinetic energy along the surface normal. Data is shown from Jansen and Juurlink [5] (squares) and from velocity-resolved kinetics (VRK, crosses). Experimental VRK data is on an arbitrary axis and was scaled here for agreement with Jansen and Juurlink's King and Wells data [5]. We use the VRK data to determine energy dependence of the sticking coefficient, equation (S9).

S2. Optimized Lattice Constants using Density Functional Theory

We optimized the lattice constant of bulk platinum using density functional theory with five different exchange-correlation functionals. The resulting values, listed in Table S1, were used to generate the bulk coordinates for the Pt(332) slab models.

Table S1: Optimized lattice constants using density functional theory with five different exchange correlation functionals (see first row) in comparison to the experiment. All values are given in Å.

RPBE	PBE	RPBE-D3	PBE-TS	optB86b-vdW	experiment [1]
3.990	3.967	3.938	3.932	3.948	3.923

S3. Experimental Rate Constants for Hydrogen (H_2) Oxidation

Figure S2 shows effective pseudo first order decay rate constants, k_{eff}, for the H_2 oxidation reaction on Pt(332) as a function of the steady state hydrogen surface coverage [H*] and surface temperature. The second order reaction rate constant, k_1, is obtained from the slope of a linear fit with fixed intercept at zero, $k_{\mathrm{eff}} = \frac{1}{2} k_1 [\mathrm{H}^*]$. Fits of this type are shown by the dashed grey lines in Figure S2. Results for k_1 are given in Figure S4(a).

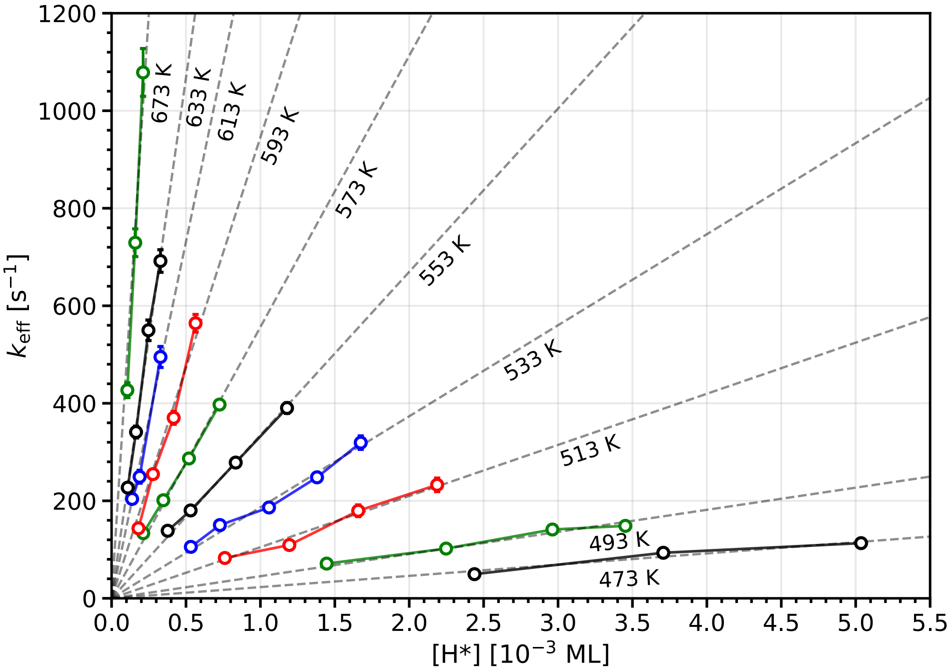

Figure S2: First order decay rate constants k_{eff} of H_2 oxidation on Pt(332) as a function of hydrogen atom surface coverage [H]. Results are shown for surface temperatures between 473 K and 673 K as indicated in the plot. Dashed lines are linear fits with fixed intercept at zero, solid lines are drawn to guide the eye.*

S4. Transition State Theory Rate Constants for O*+H*→OH*

The main text describes the calculation of transition state theory (TST) constants for the O*+H* reaction based on density functional theory (DFT). Here, we present the details of this procedure. The TST rate constant calculation requires several inputs: the zero-point energy (ZPE) corrected barrier height, which is described in subsection S4.1, the partition function of the O* atom, see subsection S4.2, and the transition state partition function, see subsection S4.3. Additionally, an accurate partition function of the H* atom—previously determined in ref. 2—is used in the calculation. For details on the H* atom partition function, refer to the main text and ref. 2.

S4.1. O*+H*→OH* Barrier Height

Table S2 shows the optimized structures of the H* atom, the O* atom, the [O···H]* transition state and the Pt(332) slab, together with classical energies and harmonic vibrational zero-point energies computed using DFT with 5 different exchange-correlation functionals. Here, we define the classical energy as the energy obtained directly from DFT calculations, without applying zero-point corrections.

Table S2: Density functional theory calculations for the O+H* barrier height using five different exchange correlation functionals (see first row). The first column labels the structures; the second column shows optimized geometries. Pt atoms are shown in grey, with step Pt atoms highlighted in blue. Oxygen atoms are colored red and hydrogen atoms white. In the other cells, the first numerical value refers to the classical energy ε of the respective structure, the second and third values (if given) are harmonic zero-point energies (ZPE) of the adsorbates for the H and D isotope, respectively. All values are given in eV.*

		RPBE	PBE	RPBE-D3	PBE-TS	optB86b-vdW
H*	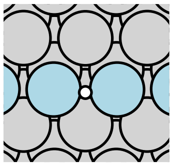	-501.946, 0.161, 0.113	-559.440, 0.161, 0.114	-579.311, 0.163, 0.115	-595.591, 0.161, 0.114	-332.843, 0.158, 0.112
O*	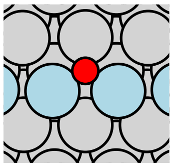	-503.991, 0.065	-561.849, 0.068	-581.395, 0.066	-598.002, 0.069	-333.769, 0.069
[O···H]*	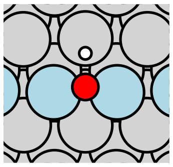	-507.010, 0.197, 0.157	-564.887, 0.200, 0.159	-584.586, 0.198, 0.157	-601.132, 0.201, 0.160	-336.718, 0.200, 0.159
slab	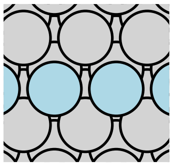	-498.148	-555.606	-575.255	-591.684	-329.083

From these calculations, we determine the classical barrier $\varepsilon_{\mathrm{O^*+H^*}}$ for OH* formation as

$$\varepsilon_{\mathrm{O^*+H^*}} = \varepsilon_{\mathrm{[O\cdots H]^*}} - \varepsilon_{\mathrm{H^*}} - \varepsilon_{\mathrm{O^*}} + \varepsilon_{\mathrm{slab}}\,, \tag{S10}$$

and the ZPE-corrected barrier as

$$E_{\mathrm{O^*+H^*}} = \varepsilon_{\mathrm{O^*+H^*}} + \mathrm{ZPE}_{\mathrm{[O\cdots H]^*}} - \mathrm{ZPE}_{\mathrm{H^*}} - \mathrm{ZPE}_{\mathrm{O^*}}\,. \tag{S11}$$

Similarly, the value for $E_{\mathrm{O^*+D^*}}$ is obtained using ZPEs for the deuterium isotopologues. Resulting barriers are given in Table 3 of the main text.

S4.2. Oxygen Atom Partition Function

We calculate the partition function of adsorbed oxygen atoms on Pt(332) using a modified harmonic oscillator partition function [10], which accounts for multiple adsorption sites within the Pt(332) unit cell. See also equation (2) of the main text. We optimized various geometries of adsorbed oxygen atoms on Pt(332) using DFT and found 7 adsorption geometries with ZPE corrected energies ≤0.5 eV above the energy of the most stable adsorption site. Table S3 shows all 7 optimized adsorption geometries, with number 1 being the most stable adsorption site. Table S4 lists ZPE corrected energies of the adsorption geometries obtained with five different exchange-correlation functionals. Table S5 shows harmonic frequencies of the oxygen atoms in the respective adsorption sites.

Table S3: Optimized geometries of O atoms adsorbed on Pt(332) based on density functional theory (DFT). Pt atoms are shown in grey, with step Pt atoms highlighted in blue. Oxygen atoms are colored red. The dashed white lines indicate cell boundaries in DFT calculations. Energies and frequencies of these structures are shown in Table S4 and Table S5, respectively.*

1 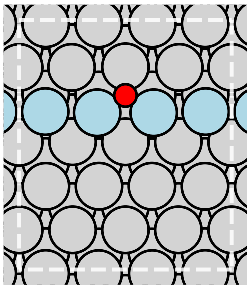	2 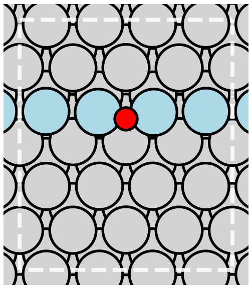	3 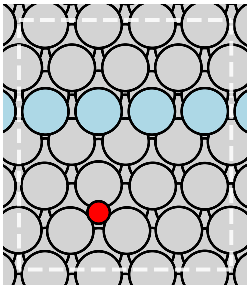	4 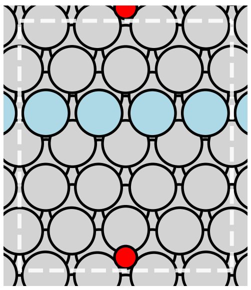
5 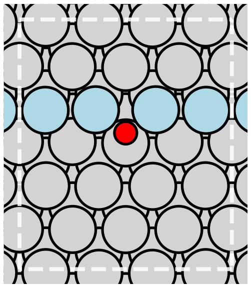	6 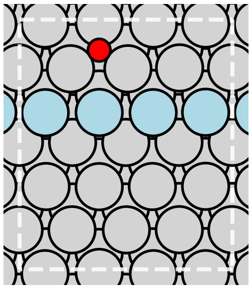	7 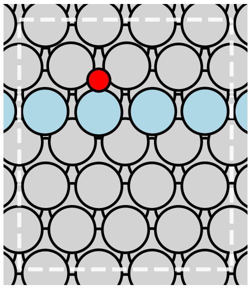	

Table S4: Vibrational zero-point corrected energies (in eV) of the oxygen adsorption geometries shown in Table S3, referenced to the most stable adsorption site (number 1). Values were obtained using density functional theory using five exchange-correlation functionals as indicated in the first row.

structure	RPBE	PBE	RPBE-D3	PBE-TS	optB86b-vdW
1	0.000	0.000	0.000	0.000	0.000
2	0.063	0.094	0.045	0.077	0.135
3	0.288	0.296	0.323	0.328	0.289
4	0.318	0.326	0.333	0.354	0.315
5	0.330	0.338	0.393	0.394	0.349
6	0.377	0.388	0.401	0.417	0.383
7	0.427	0.439	0.411	0.466	0.445

Table S5: Harmonic vibrational frequencies (in cm^{-1}) of the adsorption geometries shown in Table S3. Values were obtained using density functional theory using five exchange-correlation functionals as indicated in the first row.

structure	RPBE	PBE	RPBE-D3	PBE-TS	optB86b-vdW
1	426, 334, 295	498, 413, 118	439, 357, 342	437, 351, 331	393, 392, 307
2	440, 346, 308	503, 425, 117	452, 369, 354	449, 362, 341	403, 399, 318
3	432, 328, 297	504, 407, 115	443, 359, 337	442, 349, 320	394, 380, 306
4	452, 350, 303	514, 422, 122	457, 362, 352	457, 360, 340	415, 397, 308
5	446, 349, 318	508, 428, 115	458, 378, 361	452, 364, 346	406, 403, 327
6	426, 334, 295	498, 413, 118	439, 357, 342	437, 351, 331	393, 392, 307
7	440, 346, 308	503, 425, 117	452, 369, 354	449, 362, 341	403, 399, 318

S4.3. [O···H]* Transition State Partition Function

The partition function of the [O···H]* transition state is calculated with equation (2) of the main text, where ν are the real harmonic frequencies of the transition state. These frequencies are computed from DFT using five different exchange-correlation functionals; results are listed in Table S6. This table also shows the imaginary transition state frequencies, which we denote with "i". Figure S3 shows the transition state geometry and indicates the reaction coordinate with arrows. Note that the reaction coordinate largely involves H-atom motion towards the oxygen atom.

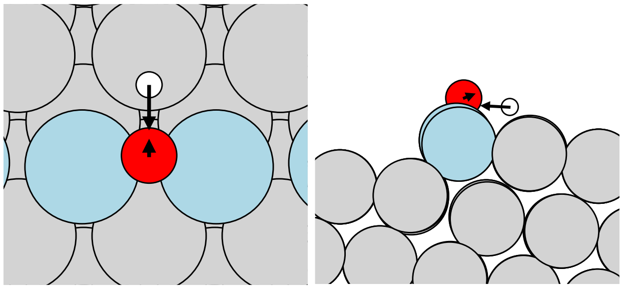

Figure S3: Transition state geometry with top and side view. Pt atoms are shown in grey, with step Pt atoms highlighted in blue. Oxygen atoms are colored red and hydrogen atoms white. The black arrows indicate displacements along the imaginary mode of the transition state.

Table S6: Harmonic vibrational frequencies of the O+H* and O*+D* transition state at the atomic step sites of Pt(332). Values are based on density functional theory calculations using five different exchange-correlation functionals and given in cm^{-1}.*

	[O···H]*	[O···D]*
RPBE	1544, 628, 475, 309, 218, 910i	1098, 470, 445, 309, 207, 684i
PBE	1562, 625, 489, 324, 220, 908i	1110, 483, 443, 324, 209, 682i
RPBE-D3	1563, 630, 475, 295, 229, 929i	1111, 469, 447, 294, 218, 697i
PBE-TS	1571, 620, 498, 323, 224, 895i	1116, 492, 440, 322, 212, 673i
optB86b-vdW	1542, 637, 492, 328, 220, 938i	1096, 486, 452, 327, 209, 702i

S4.4. Comparison to Experimental Rate Constants

We use velocity-resolved kinetics data to determine the rate constants k_{eff} of the hydrogen oxidation reaction. Equation (9) of the main text relates k_{eff} to the elementary rate constant k_1 of the rate determining step via $k_1 = 2k_{\text{eff}}/[\text{H}^*]$. Figure S4 shows k_1 determined from experiment (squares) and transition state theory (lines) employing five different exchange correlation functionals for the computation of barrier heights and partition functions. Panel (a) shows results for O*+H* and panel (b) for O*+D*.

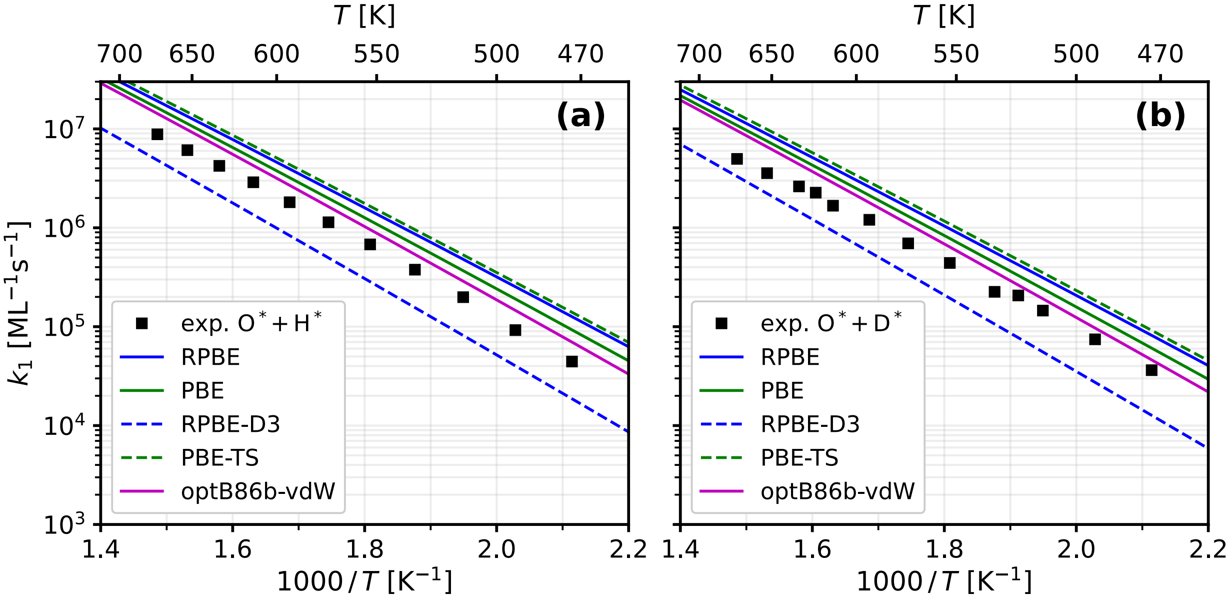

Figure S4: Second order rate constants for the rate determining step of hydrogen oxidation reaction, O+H* (a) and O*+D* (b), determined from experiment (squares) and from transition state theory (TST). TST predictions are shown based on DFT data obtained with five different exchange correlation functionals as indicated in the legend.*

S4.5. Effect of the Static Surface Approximation in Partition Function Calculations

In this work, we calculated partition functions of adsorbates on a frozen metal surface. However, this is an approximation that is only valid, if the adsorbate partition function is decoupled from the metal's partition function, and if the partition function associated with the metal does not change significantly between the transition state (TS) and the initial state (IS). In such cases, the effect of the metal's degrees of freedom on the TST rate constant cancels out because only the ratio of the TS and IS partition functions is relevant to the TST rate expression—see also equation (1) of the main text.

To test this approximation, we computed frequencies of the entire Pt slab (excluding the rigid bottom layer) with the adsorbates on it for both the transition state and the initial state. Then, we computed the harmonic oscillator partition function ratio $Q_{\mathrm{TS}}/Q_{\mathrm{IS}}$ of this combined system and compared it with the results obtained by considering only the adsorbate frequencies on a frozen Pt surface. Results are shown in Figure S5.

We find that the adsorbate frequencies do not change significantly when including the metal's degrees of freedom (DOF), as expected due to the large mass disparity between the adsorbate and the metal. Additionally, the metal's frequencies do not change between TS and IS, see panel (a) of Figure S5. These observations validate the approximations presented above. Figure S5(b) shows the resulting error made by neglecting the metal's degrees of freedom when computing the partition function ratio and, consequently, the TST rate constants. This error is below 1.5% within the studied temperature range.

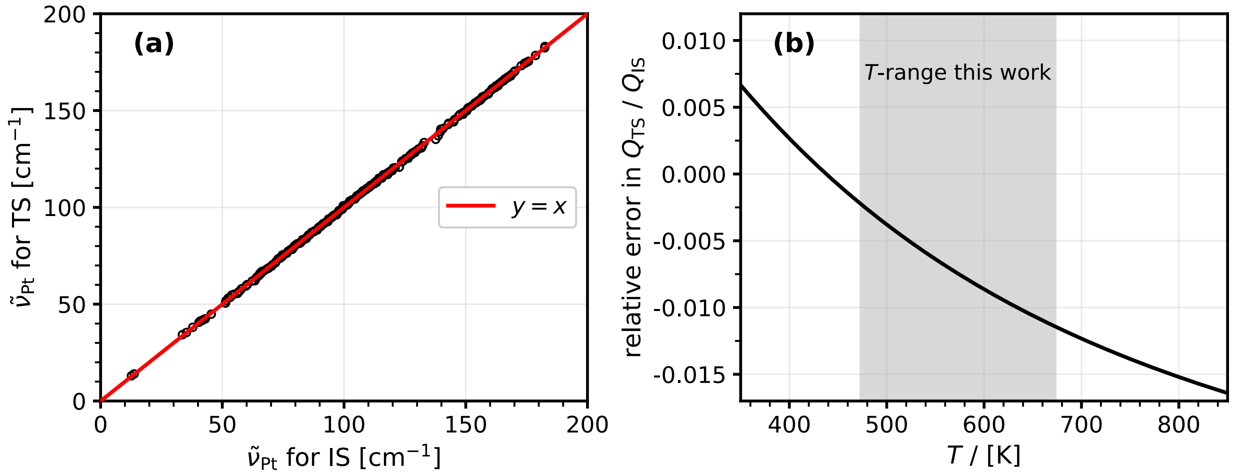

Figure S5: Effect of the static surface approximation in partition function calculations for the O+H*→OH* reaction. Panel (a) shows that the metal frequencies change only insignificantly between the transition state (TS) for OH* formation and the initial state (IS). Panel (b) shows the relative error made by excluding the metal's frequencies in the calculation of the partition function ratio that enters the transition state theory rate expression.*

Although treating the metal's frequencies as harmonic oscillators is not exact, we believe this approach provides a reasonable estimate of the error made in computing the TST rate constant. It is important to note that the system studied here may be particularly favorable for neglecting the metal's DOF, since the adsorbates are light and most of the structural change between IS and TS derives from H atom motion. We expect larger errors for reactions involving heavier atoms, larger structural rearrangements, and, especially, desorption processes, as pointed out in previous work [11].

S5. Transition State Theory Rate Constants for $OH^*+H^*\rightarrow H_2O^*$ and $2OH^*\rightarrow H_2O^*+O^*$

Here we present details of the calculation of transition state theory (TST) constants for the OH*+H* and OH*+OH* reaction based on density functional theory (DFT). The TST rate constant calculation requires several inputs: the zero-point energy (ZPE) corrected barrier height, which is described in subsection S5.1, the partition function of the OH* adsorbate, see subsection S5.2, and the transition state partition functions, see subsection S5.3. Additionally, an accurate partition function of the H* atom—previously determined in ref. 2—is used in the calculation. For details on the H* atom partition function, refer to the main text and ref. 2.

S5.1. $OH^*+H^*\rightarrow H_2O^*$ and $2OH^*\rightarrow H_2O^*+O^*$ Barrier Heights

Table S7 shows the optimized structures of the H* atom, the OH* adsorbate, the [OH···H]* and [OH···OH]* transition states and the Pt(332) slab, with classical energies and harmonic vibrational zero-point energies.

Table S7: Density functional theory calculations for OH+H* and OH*+OH* barrier height using the RPBE and PBE exchange correlation functional. The first column labels the structures; the second column shows optimized geometries. Pt atoms are shown in grey, with step Pt atoms highlighted in blue. Oxygen atoms are colored red and hydrogen atoms white. In the other cells, the first numerical value refers to the classical energy ε of the respective structure, the second and third values (if given) are harmonic zero-point energies (ZPE) of the adsorbates for the H and D isotope, respectively. All values are given in eV.*

		RPBE	PBE
H*	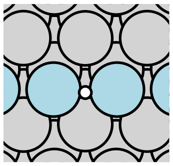	-501.946, 0.161, 0.113	-559.440, 0.161, 0.114
OH*	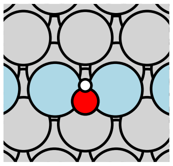	-508.269, 0.354, 0.267	-566.075, 0.357, 0.271
[OH···H]*	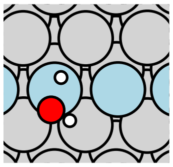	-511.227, 0.487, 0.361	-568.990, 0.488, 0.362
[OH···OH]*	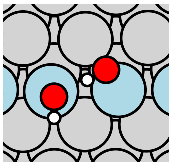	-518.117, 0.698, 0.530	-576.235, 0.699, 0.531
slab	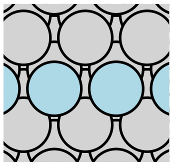	-498.148	-555.606

From these calculations, we determine the classical barrier heights as

$$\varepsilon_{\mathrm{OH}^*+(\mathrm{O})\mathrm{H}^*} = \varepsilon_{[\mathrm{OH}\cdots(\mathrm{O})\mathrm{H}]^*} - \varepsilon_{(\mathrm{O})\mathrm{H}^*} - \varepsilon_{\mathrm{OH}^*} + \varepsilon_{\mathrm{slab}}\,, \tag{S12}$$

and the ZPE corrected barrier height as

$$E_{\mathrm{OH}^*+(\mathrm{O})\mathrm{H}^*} = \varepsilon_{\mathrm{OH}^*+(\mathrm{O})\mathrm{H}^*} + \mathrm{ZPE}_{[\mathrm{OH}\cdots(\mathrm{O})\mathrm{H}]^*} - \mathrm{ZPE}_{(\mathrm{O})\mathrm{H}^*} - \mathrm{ZPE}_{\mathrm{OH}^*}\,. \tag{S13}$$

Similarly, the value for $E_{\mathrm{OD}^*+(\mathrm{O})\mathrm{D}^*}$ is obtained using ZPEs for the deuterium isotopologues. Computed barrier heights are given in Table S8.

Table S8: Zero-point energy corrected barrier heights for the OH+H* and OH*+OH* reaction at Pt(332) steps obtained from density functional theory using the RPBE and PBE exchange correlation functional. All values are given in eV.*

	RPBE	PBE
[OH···H]*	0.81	0.89
[OD···D]*	0.82	0.90
[OH···OH]*	0.26	0.29
[OD··· OD]*	0.27	0.30

S5.2. OH* Partition Function

We calculate the partition function of adsorbed OH* on Pt(332) using a modified harmonic oscillator partition function [10], which accounts for multiple adsorption sites within the Pt(332) unit cell. See also equation (2) of the main text. We optimized various geometries of OH* on Pt(332) using DFT and found 4 adsorption geometries with ZPE corrected energies ≤0.5 eV above the energy of the most stable adsorption site. Table S9 shows all 4 optimized adsorption geometries along with their ZPE corrected energies and harmonic frequencies for the OH* adsorbates.

Table S9: Optimized geometries, energies and harmonic frequencies of the hydroxyl molecule adsorbed on the Pt(332) surface. Results were obtained with density functional theory (DFT) using the RPBE and PBE exchange correlation functional. The first row shows the adsorption geometries. Pt atoms are colored grey, with step Pt atoms highlighted in blue. Oxygen atoms are colored red and hydrogen atoms white. The dashed white lines indicate cell boundaries in DFT calculations. Vibrational zero-point corrected energies are given with respect to the most stable geometry. Harmonic vibrational frequencies are shown in the last rows.

		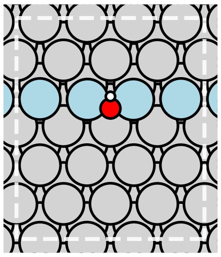	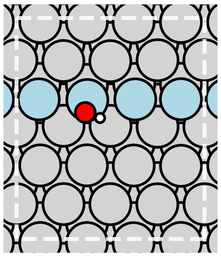	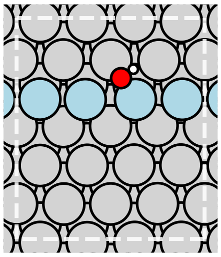	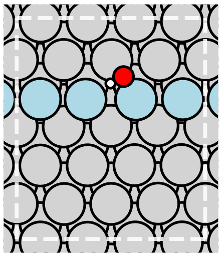
energies [eV]	RPBE	0.000	0.019	0.333	0.378
	PBE	0.000	0.089	0.322	0.369
frequencies OH* [cm^{-1}]	RPBE	3687, 710, 687, 369, 148, 103	3671, 870, 519, 216, 125, 83	3632, 732, 680, 372, 164, 152	3625, 721, 667, 368, 176, 173
	PBE	3677, 726, 683, 388, 190, 102	3668, 867, 538, 226, 119, 70	3611, 734, 695, 390, 195, 168	3604, 726, 673, 384, 187, 164
frequencies OD* [cm^{-1}]	RPBE	2682, 519, 504, 363, 143, 98	2671, 645, 504, 161, 118, 79	2642, 539, 497, 365, 159, 144	2637, 530, 489, 361, 168, 166
	PBE	2675, 531, 502, 382, 184, 97	2669, 644, 520, 168, 113, 67	2627, 539, 511, 383, 190, 159	2621, 533, 494, 376, 180, 156

S5.3. [OH···H]* and [OH···OH]* Transition State Partition Function

The partition function of the [OH···H]* and [OH···OH]* transition states are calculated employing equation (2) of the main text, where ν are the real harmonic frequencies of the transition state. These frequencies are computed from DFT with the RPBE and PBE exchange correlation functional, results are listed in Table S10. This table also shows the imaginary transition state frequencies, which we denote with "i".

Table S10: Harmonic vibrational frequencies of the OH+H* and OH*+OH* transition states, as well as their deuterium isotopologues. Values are based on density functional theory calculations using the RPBE and the PBE exchange-correlation functional and given in cm^{-1}.*

	RPBE	PBE
[OH···H]*	3674, 2034, 895, 500,<br>425, 154, 111, 66, 877i	3667, 2039, 891, 491,<br>447, 150, 109, 79, 901i
[OD···D]*	2673, 1439, 656, 421,<br>355, 120, 100, 62, 634i	2668, 1443, 654, 438,<br>355, 119, 93, 76, 650i
[OH···OH]*	3703, 3121, 1079, 883,<br>826, 487, 471, 263,<br>210, 123, 100, 119i	3710, 2964, 1122, 929,<br>822, 519, 487, 255,<br>238, 128, 100, 110i
[OD···OD]*	2694, 2277, 782, 638,<br>605, 479, 458, 203,<br>199, 116, 94, 117i	2699, 2164, 812, 670,<br>605, 511, 472, 230,<br>195, 120, 95, 108i

S6. Microkinetic Model of Hydrogen Oxidation on Pt(332)

S6.1. Construction of the Microkinetic Model

The microkinetic model of the hydrogen oxidation reaction consists of five elementary reactions given in equations (S14) to (S18). We use the time profile $F_{O_2}(t)$ of the molecular beam to initiate the sequence of reactions. At the temperatures of our work, 473 K to 673 K, water desorption, equation (S18), is several order of magnitude faster than the timescale of our kinetics experiments [12], causing an insignificant lifetime of adsorbed water. We thus do not account for reverse reactions to reactions (S16) and (S17) or the kinetics of equation (S18). The microkinetic model accounts for the possibility of OH* dissociation, the corresponding rate constant k_{-1} is computed with equation (1) of the main text using the transition state partition function as described in section S4.3 and the OH* partition function from section S5.2.

$$\mathrm{O}_{2(\mathrm{beam})} \xrightarrow{F_{\mathrm{O}_2}(t)} 2\mathrm{O}^* \tag{S14}$$

$$\mathrm{O}^* + \mathrm{H}^* \underset{k_{-1}}{\overset{k_1}{\leftrightarrows}} \mathrm{OH}^* \tag{S15}$$

$$\mathrm{OH}^* + \mathrm{H}^* \overset{k_2}{\rightarrow} \mathrm{H_2O}^* \tag{S16}$$

$$2\mathrm{OH}^* \overset{k_3}{\rightarrow} \mathrm{H_2O}^* + \mathrm{O}^* \tag{S17}$$

$$\mathrm{H_2O}^* \xrightarrow{\text{fast}} \mathrm{H_2O}_{(\mathrm{g})} \tag{S18}$$

This mechanism can be expressed with the rate equations (S19) and (S20), where $[\mathrm{H}^*]$ is the steady state hydrogen coverage of the experiment.

$$\frac{\mathrm{d}[\mathrm{O}^*]}{\mathrm{d}t} = -k_1[\mathrm{O}^*][\mathrm{H}^*] + k_{-1}[\mathrm{OH}^*] + k_3[\mathrm{OH}^*]^2 + 2F_{\mathrm{O}_2}(t) \tag{S19}$$

$$\frac{\mathrm{d}[\mathrm{OH}^*]}{\mathrm{d}t} = k_1[\mathrm{O}^*][\mathrm{H}^*] - k_2[\mathrm{OH}^*][\mathrm{H}^*] - k_{-1}[\mathrm{OH}^*] - 2k_3[\mathrm{OH}^*]^2 \tag{S20}$$

These differential equations are solved numerically using the SciPy library [13] in Python, yielding the OH* concentration $[\mathrm{OH}^*](t)$ as a function of reaction time t. The water formation rate $F_{\mathrm{H_2O}}(t)$ is computed from equation (S21).

$$F_{\mathrm{H_2O}}(t) = k_2[\mathrm{H}^*][\mathrm{OH}^*](t) + k_3([\mathrm{OH}^*](t))^2 \tag{S21}$$

The microkinetic model was evaluated using rate constants for reactions at the step sites of the Pt(332) surface, derived in sections S4 and S5, and for reactions on the (111) terraces of the Pt(332) surface. Both results are shown in Figure (2) of the main text. We approximated rate constants for the Pt(332) terrace with previously reported rate constants for Pt(111) [14], corrected by the step-terrace energy difference of the O* adsorbate (0.29 eV) and the OH* adsorbate (0.54 eV). These values were derived from DFT and agree for the PBE and RPBE functional.

S6.2. Degree of Rate Control Analysis

We use a degree of rate control (DRC) [15] analysis to determine the rate determining step of the hydrogen oxidation reaction. The DRC is defined in equation (S22) as the relative change in water formation rate F_{H_2O} per relative change in the rate constant k_i of an elementary step i, while all other rate constants $k_{i\neq j}$ and all equilibrium constants K_i are kept fixed [15]. Since the DRC method was designed primarily for analysis of steady-state kinetics, we have used a variant of the DRC method for transient kinetics [16].

$$\mathrm{DRC} = \frac{k_i}{F_{H_2O}}\left(\frac{\partial F_{H_2O}}{\partial k_i}\right)_{k_{j\neq i},K_i} \tag{S22}$$

Figure S6 is similar to Figure (2) of the main text and shows peak normalized deuterium oxidation rates based on PBE rate constants (black lines) as a function of surface deuterium coverage and temperature. The red, blue, green lines are the DRCs for $k_i = k_1, k_2, k_3$, respectively. This analysis clearly shows negligible contributions of reactions (S16) and (S17) to the DRC at all relevant reaction times, from which we can conclude that reaction (S15) is the only rate determining step in this mechanism. Slight deviations from this behavior can be seen when the water formation rate levels off. In these cases, the OD* concentration is low, which slows down reactions (S16) and (S17) and their reaction rates become comparable to the rate of reaction (S15).

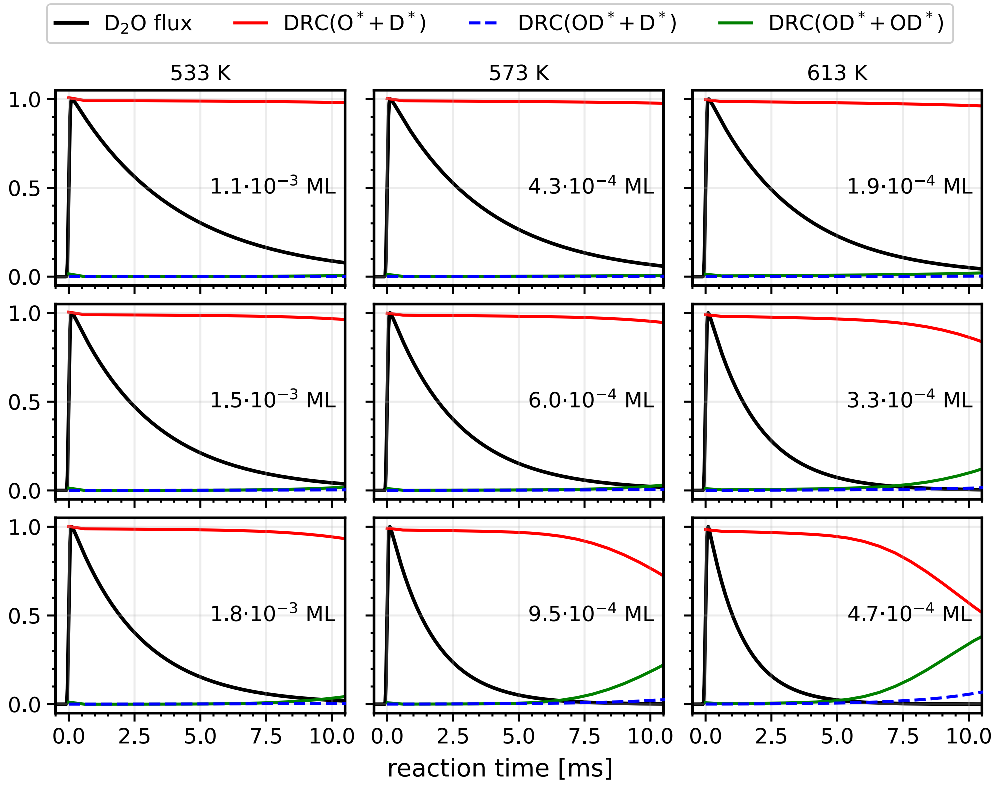

Figure S6: Degree of rate control (DRC) analysis for the deuterium oxidation reaction on Pt(332). Black lines are peak normalized kinetic traces from a microkinetic model based on PBE rate constants. The red, blue and green lines are the DRCs of the O+D*, the OD*+D* and the OD*+OD* reaction rate constants, respectively. Steady state deuterium coverages are given in each panel. Surface temperatures, given at the top of the columns, apply to all panels within a column.*

S7. Additional References

1 Leontyev, I. N. *et al.* Size dependence of the lattice parameters of carbon supported platinum nanoparticles: X-ray diffraction analysis and theoretical considerations. *RSC Advances* **4**, 35959-35965 (2014). https://doi.org/10.1039/C4RA04809A

2 Borodin, D. *et al.* Quantum effects in thermal reaction rates at metal surfaces. *Science* **377**, 394-398 (2022). https://doi.org/doi:10.1126/science.abq1414

3 Borodin, D. *et al.* NO Binding Energies to and Diffusion Barrier on Pd Obtained with Velocity-Resolved Kinetics. *The Journal of Physical Chemistry C* **125**, 11773-11781 (2021). https://doi.org/10.1021/acs.jpcc.1c02965

4 Fingerhut, J. *et al.* Binding Energy and Diffusion Barrier of Formic Acid on Pd(111). *The Journal of Physical Chemistry A* **127**, 142-152 (2023). https://doi.org/10.1021/acs.jpca.2c07414

5 Jansen, C. & Juurlink, L. Absolute dissociation cross sections for D_2 dissociation on Pt steps. *Chemical Physics Letters* **776**, 138679 (2021). https://doi.org/10.1016/j.cplett.2021.138679

6 Luntz, A. C., Brown, J. K. & Williams, M. D. Molecular beam studies of H_2 and D_2 dissociative chemisorption on Pt(111). *The Journal of Chemical Physics* **93**, 5240-5246 (1990). https://doi.org/10.1063/1.459669

7 Gee, A. T., Hayden, B. E., Mormiche, C. & Nunney, T. S. The role of steps in the dynamics of hydrogen dissociation on Pt(533). *The Journal of Chemical Physics* **112**, 7660-7668 (2000). https://doi.org/10.1063/1.481360

8 van Lent, R. *et al.* Site-specific reactivity of molecules with surface defects—the case of H_2 dissociation on Pt. *Science* **363**, 155-157 (2019). https://doi.org/10.1126/science.aau6716

9 Nitz, F. *et al.* Thermal Rates and High-Temperature Tunneling from Surface Reaction Dynamics and First-Principles. *Journal of the American Chemical Society* **146**, 31538-31546 (2024). https://doi.org/10.1021/jacs.4c09017

10 Blöndal, K., Sargsyan, K., Bross, D. H., Ruscic, B. & Goldsmith, C. F. Configuration Space Integration for Adsorbate Partition Functions: The Effect of Anharmonicity on the Thermophysical Properties of CO–Pt(111) and CH_3OH–Cu(111). *ACS Catalysis* **13**, 19-32 (2023). https://doi.org/10.1021/acscatal.2c04246

11 Li, C., Li, Y. & Jiang, B. First-principles surface reaction rates by ring polymer molecular dynamics and neural network potential: role of anharmonicity and lattice motion. *Chemical Science* **14**, 5087-5098 (2023). https://doi.org/10.1039/D2SC06559B

12 Picolin, A., Busse, C., Redinger, A., Morgenstern, M. & Michely, T. Desorption of H_2O from Flat and Stepped Pt(111). *The Journal of Physical Chemistry C* **113**, 691-697 (2009). https://doi.org/10.1021/jp808170f

13 Virtanen, P. *et al.* SciPy 1.0: fundamental algorithms for scientific computing in Python. *Nature Methods* **17**, 261-272 (2020). https://doi.org/10.1038/s41592-019-0686-2

14 Borodin, D. *et al.* The puzzle of rapid hydrogen oxidation on Pt(111). *Molecular Physics* **119**, e1966533 (2021). https://doi.org/10.1080/00268976.2021.1966533

15 Campbell, C. T. The Degree of Rate Control: A Powerful Tool for Catalysis Research. *ACS Catalysis* **7**, 2770-2779 (2017). https://doi.org/10.1021/acscatal.7b00115

16 Campbell, C. T. Finding the Rate-Determining Step in a Mechanism: Comparing DeDonder Relations with the "Degree of Rate Control". *Journal of Catalysis* **204**, 520-524 (2001). https://doi.org/https://doi.org/10.1006/jcat.2001.3396
